# Cross-Kingdom Enzymatic Strategies for Deoxynivalenol Detoxification: Computational Analysis of Structural Mechanisms and Evolutionary Adaptations

**DOI:** 10.3390/microorganisms13102384

**Published:** 2025-10-16

**Authors:** Francisco J. Enguita, Ana Lúcia Leitão

**Affiliations:** 1 Faculdade de Medicina, Universidade de Lisboa, Av. Prof. Egas Moniz, 1649-028 Lisboa, Portugal; fenguita@medicina.ulisboa.pt; 2 Departamento de Química, Faculdade de Ciências e Tecologia, Campus da Caparica, Universidade NOVA de Lisboa, 2829-516 Caparica, Portugal

**Keywords:** deoxynivalenol, *Fusarium*, biodegradation, detoxification, structural biology, docking, molecular dynamics, evolution

## Abstract

Deoxynivalenol (DON) is a trichothecene mycotoxin produced by *Fusarium* species that frequently contaminates cereal crops, representing a major threat to food safety, public health, and agricultural productivity. Its remarkable chemical stability during food processing presents significant challenges for effective detoxification. Among the available mitigation strategies, biological approaches have emerged as particularly promising, as they exploit enzymatic systems capable of converting DON into metabolites with substantially reduced toxicity. In this study, we provide a comprehensive analysis of the structural and evolutionary mechanisms underlying DON detoxification across three kingdoms of life. We investigated the fungal glutathione S-transferase Fhb7, the bacterial DepA/DepB epimerization pathway, and the plant SPG glyoxalase using integrative bioinformatics, phylogenetics, molecular modeling, and docking simulations. The selected enzymatic systems employ distinct yet complementary strategies: Fhb7 conjugates DON with glutathione and disrupts its epoxide ring, DepA/DepB converts it into the less toxic 3-epi-DON through stereospecific epimerization, and SPG glyoxalase mediates DON isomerization. Despite their mechanistic differences, these enzymes share key adaptive features that enable efficient DON recognition and detoxification. This work provides an integrative view of cross-kingdom enzymatic strategies for DON degradation, offering insights into their evolution and functional diversity. These findings open avenues for biotechnological applications, including the development of DON-resistant crops and innovative solutions to reduce mycotoxin contamination in the food chain.

## 1. Introduction

Deoxynivalenol (DON) is a secondary metabolite, belonging to the group of terpenoid mycotoxins. It is a contaminant of many crops, mainly wheat, maize, barley, rice, rye and oats, being the most commonly occurring type B trichothecene [1,2]. This mycotoxin, also known as vomitoxin for its capacity to induce vomiting in various mammalian species, is produced by several species of the genera *Fusarium*, including *F. graminearum*, *F. culmorum*, *F. sambucinum*, *F. roseum* and *F. crookwellense*, that could contaminate the grain during postharvest procedures and storage [1,3,4]. Structurally, with the chemical name 12,13-epoxy-3α,7α,15-trihydroxytrichothec-9-en-8-one, this polar compound is stable during food processing under high pressure, heat and acidic environment [5,6]. DON is detected not only in raw materials, but also in processed products such as wheat flour, biscuits, cakes, bread, snacks, pasta, pizzas, breakfast cereals among others [7,8].

The International Agency for Research on Cancer (IARC) has classified DON as a Group 3 agent, indicating that its carcinogenicity in humans cannot be determined [9]. According to Samet and colleagues, a classification as Group 3 does not imply evidence of non-carcinogenicity or general safety [10]. In fact, there are several studies of the negative effect of DON in the human cell lines (Caco-2 cells—[11]; U937 cells—[12]; HepG2 cells—[13]; and lymphocytes—[14]). It is well established that DON primarily targets the ribosomes, mitochondria, and lysosomes. This mycotoxin also triggers oxidative stress by elevating reactive oxygen species (ROS) levels and promoting lipid peroxidation, while simultaneously suppressing protein and DNA synthesis and ultimately leading to cell death [11,12,13]. Moreover, even at a low concentration, DON showed high potential to modify gene expression through the alteration of splicing patterns and mRNA stability [15].

Human gastroenteritis with nausea, diarrhea or vomiting as primary symptoms were frequently associated with DON ingestion [2]. A gastroenteritis outbreak in India associated with the consumption of rain-damaged moldy wheat products was reported [16]. Even though all age-groups were affected by illness, in children who had consumed substantial amounts of wheat preparations over periods of more than a week, upper respiratory tract infections were also described [16]. Recently, an outbreak caused by DON-contaminated commercial packaged noodles in a primary school in China was reported, being the estimated exposure doses of the reported cases significantly above the tolerable daily intake (TDI) [17]. The possibility of secondary infections and the relatively higher food intake per kg of body weight indicate that special attention should be given in the case of young children. This scenario may be further exacerbated when considering co-exposure to multiple mycotoxins. A study conducted on the Portuguese general population between 2015 and 2016 revealed measurable exposure to six mycotoxins, including DON, zearalenone (ZEN), ochratoxin A (OTA), and fumonisin B1 (FB1), the latter two of which are classified by the IARC as possibly carcinogenic to humans. Furthermore, risk characterization indicated that exposure of the Portuguese population to DON, OTA and FB1 was above safety limits [18]. Although predicting the combined effects of co-occurring mycotoxins is challenging, potential additive and synergistic interactions may pose a significant health concern [19,20]. In humans DON is mainly absorbed in the gastrointestinal tract, distributed, metabolized and excreted in the urine [21]. A systematic investigation of the DON exposure for a larger study population performed with young Germany adults from 1996 to 2021 found higher incidence of DON in urine samples (99%) [22]. The same pattern was observed in a study conducted by Brera and coworkers, where almost all participants from Norway (99%) and UK (93%) were exposed to DON [23].

**Figure 1:**
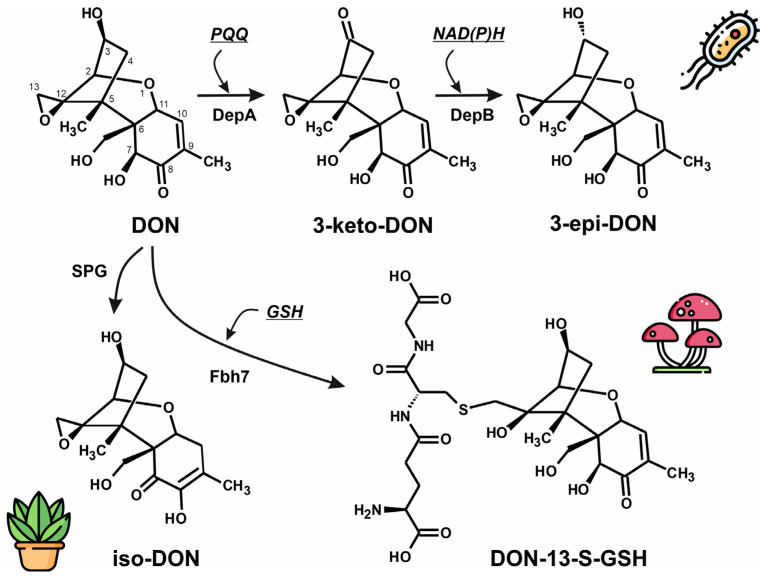
Microbial pathways for the detoxification of deoxynivalenol (DON) in bacteria, fungi and plants. In bacteria such as Devosia, toxicity of DON is reduced by the formation of its stereoisomer 3-epi-DON. This is a two-step process, where DON is firstly oxidized to generate the 3-keto-DON intermediate by a quinone-dependent oxidoreductase encoded by depA gene. The keto intermediate will be later converted into 3-epi-DON, by an oxidoreductase encoded by the depB gene. In fungi as *Epichloë* or *Aspergillus*, DON can be detoxified by conjugation to glutathione by Fbh7 enzyme. An additional mechanism for DON detoxification was also described in cotton plants, involving a Zn-dependent glyoxalase (SPG glyoxalase) that produces a low-toxicity DON isomer, iso-DON. (GSH, glutathione; PQQ, pyrroloquinoline quinone; NAD, nicotinamide adenine dinucleotide).

Several strategies for the detoxification of DON have been proposed, including physical, chemical, and biological methods. Among these, biological approaches have attracted particular interest due to their relatively low resource demands, minimal impact on the quality and nutritional value of raw materials, and the production of non-toxic or low-toxicity metabolites through the action of microorganisms or enzymes during the detoxification process [24]. Microbial pathways for the detoxification of deoxynivalenol (DON) span multiple kingdoms—bacteria, fungi, and plants—each leveraging distinct enzymatic strategies to reduce toxin burden (Figure 1). In bacteria such as *Devosia mutans* 17-2-E-8, DON undergoes stereochemical inversion at its C3 position via a two-step ‘Dep’ pathway. The PQQ-dependent dehydrogenase DepA oxidizes DON to 3-keto-DON, which is then stereospecifically reduced by the NADPH-dependent aldo-keto reductase DepB to form 3-epi-DON, a metabolite with dramatically reduced toxicity, estimated to be several hundred times lower than that of DON [25]. Parallel epimerization pathways have been documented in other bacterial strains, such as *Devosia* sp. D6-9 and *Sphingomonas* sp. S3-4, featuring analogous oxidoreductases (e.g., QDDH, AKR13B2, AKR6D1) [26]. In fungi, including endophytic *Epichloë* species, the enzyme Fhb7 functions as a glutathione S-transferase that catalyzes the opening of DON epoxide ring, thereby detoxifying it and contributing to *Fusarium* head blight resistance in crops [27]. In plants such as cotton, a Zn-dependent glyoxalase (SPG glyoxalase) has been shown to isomerize DON into iso-DON, yielding a significantly less toxic outcome [28]. These complementary strategies, bacterial epimerization, fungal conjugation, and plant isomerization, highlight the evolutionary ingenuity across life forms and underscore promising routes for developing biotechnological interventions to mitigate DON contamination.

In this work, we aimed to investigate the structural and evolutionary mechanisms that underline deoxynivalenol (DON) detoxification across bacteria, fungi, and plants, with a particular focus on the enzymatic systems Fhb7, DepA/DepB, and SPG glyoxalase. By integrating phylogenetics, molecular modeling, docking, and molecular dynamics simulations, we provide a comparative framework to understand how distinct kingdoms of life have evolved with complementary strategies to counteract DON toxicity. Our analyses reveal both convergent and divergent features in catalytic architectures, highlighting common principles of substrate recognition as well as inferring possible lineage-specific adaptations.

## 2. Materials and Methods

### 2.1. Sequence Alignment and Phylogenetic Analysis

Canonical protein sequences were retrieved from UniProt database and queried against the NCBI non-redundant protein sequence database using BlastP [29], imposing an **E**-value threshold of 1 × 10^−5^ to ensure high-confidence hits. Amino acid sequences were input in FASTA format, and alignments were evaluated based on sequence identity, query coverage, and alignment scores. Output files were parsed to extract relevant homologous proteins, which were ranked by statistical significance (E-value) and alignment score. Where necessary, taxonomic filters were applied to focus on specific organisms or groups of interest, aiding downstream analyses. The aligned sequences were subsequently utilized for the construction of a phylogenetic tree using the Maximum Likelihood (ML) method. Model selection for the ML analysis was conducted by employing the model test functionality in MEGA software (Version 12) [30], selecting the substitution model with the lowest Bayesian Information Criterion (BIC) score. The reliability of the inferred tree was assessed using bootstrap analysis with 1000 replicates. Phylogenetic trees were visualized and edited within the MEGA environment to facilitate interpretation of evolutionary relationships among the analyzed sequences.

### 2.2. Residue Functional Conservation and Coevolution Analysis

Functional residues in the selected enzymes were identified using the ConSurf algorithm, which evaluates the evolutionary conservation of amino acids based on sequence alignments and phylogenetic relationships [31]. The target protein sequences were analyzed using homologs retrieved from the UniProt database, ensuring sufficient sequence diversity for reliable conservation estimation. Conservation scores were calculated using an empirical Bayesian method implemented in ConSurf, integrating phylogenetic data to identify highly conserved residues. Conserved residues were mapped onto the three-dimensional structure of the selected proteins to identify potential functional sites, including active or binding regions.

Coevolutionary relationships between residues in protein sequences were analyzed using the Mutual Information (MI) algorithm implemented in the MISTIC2 software (version 2.0) platform [32]. MISTIC2 computes MI values to quantify the statistical dependency between pairs of amino acid positions within a multiple sequence alignment (MSA), thereby identifying potential coevolving residues. Protein sequences of interest were obtained from Uniprot, and a multiple sequence alignment (MSA) was generated using MAFFT (v.7) using default parameters [33]. The alignment was curated to remove sequences with high redundancy or excessive gaps to ensure a representative and high-quality dataset. Sequence identity thresholds were set at 90% to balance sequence diversity and alignment quality. The coevolutionary relationships were visualized as a Circos diagram generated by the MISTIC2 software, highlighting high MI value pairs that potentially indicate functional or structural interdependence between residues.

### 2.3. Normal Mode Analysis

Normal mode analysis (NMA) was performed using the iModS (internal coordinates-based Molecular Dynamics and normal Modes for Structures) protocol to investigate the conformational flexibility of the protein structures [34]. iModS integrates elastic network modeling and normal mode calculations to efficiently explore large-scale structural dynamics. Protein structures were prepared by removing any heteroatoms, ions, and water molecules to focus solely on the macromolecular backbone. The iModS workflow uses a coarse-grained representation of the protein, where each residue is represented by a single alpha carbon (Cα) to construct an elastic network model. An energy-minimized structure was used as input, ensuring convergence of the normal modes to biologically relevant motions. The protocol computes the low-frequency modes, which are associated with collective motions essential for functional transitions. Analysis focused on the first six non-trivial modes, excluding translational and rotational components. Visualization and interpretation of the mode vectors were conducted to identify key conformational changes and potential functional pathways. All simulations were performed using default parameters unless otherwise specified.

### 2.4. Molecular Docking

Ligand binding sites in enzyme structures were predicted by blind docking with the CB-Dock2 algorithm [35]. CB-Dock2 identifies potential binding cavities and evaluates ligand poses based on docking scores, leveraging a curvature-based cavity detection approach integrated with AutoDock Vina scoring functions [36]. The three-dimensional structures of the enzymes retrieved from PDB database were prepared by removing ligand and water molecules and adding missing hydrogen atoms [37]. CB-Dock2 automatically detected potential binding sites within the enzyme structure and performed docking simulations for each cavity. Docking results were ranked based on binding affinities, and the top-scoring poses were analyzed to assess binding interactions, including hydrogen bonding, hydrophobic contacts, and other key interactions. Default parameters of CB-Dock2 were used, with adjustments made for grid box size and resolution if necessary. Prediction of binding affinity between DON and the selected proteins within the docked complex was estimated by the PRODIGY algorithm [38].

### 2.5. Molecular Dynamics Simulations and Free Energy Calculations

Full-atom molecular dynamics (MD) simulations were performed with GROMACS version 2025.1 in an aqueous environment [39]. The systems were solvated with explicit TIP3P water, ensuring a minimum extension of 15 Å beyond the solute along the Z-axis, and ionized with Na^+^ and Cl^−^ to achieve a physiological ionic strength of 0.15 M. Force field parameters were assigned using the CHARMM36m force field [40]. Energy minimization was performed using the steepest descent algorithm until the maximum residual force on any atom dropped below 1000 kJ·mol^−1^·nm^−1^. Subsequently, a six-step equilibration protocol provided by CHARMM-GUI was applied under NVT and NPT ensembles, gradually releasing positional restraints to stabilize the system. Production MD simulations were then executed under the NPT ensemble for 100 ns, employing periodic boundary conditions. The system temperature was maintained at 310 K using the Nosé–Hoover thermostat, while pressure was controlled semi-isotropically at 1 bar using the Parrinello–Rahman barostat. Long-range electrostatics were treated using the Particle Mesh Ewald (PME) method with a real-space cutoff of 1.2 nm. All covalent bonds involving hydrogen atoms were constrained using the LINCS algorithm, allowing for a 2 fs integration time step. Trajectory coordinates and system energies were saved at 10 ps intervals for subsequent analysis.

The binding free energy (ΔG_bind) of protein–ligand complexes was estimated using the Molecular Mechanics Poisson–Boltzmann Surface Area (MM-PBSA) approach, as implemented in gmx_MMPBSA v1.5.7 [41]. A single-trajectory protocol was employed to minimize conformational variability by computing the molecular mechanics energies of the protein, ligand, and complex from identical MD frames. The 100 ns MD trajectories were sampled into 1000 representative snapshots and the calculations were performed in triplicate. For each snapshot, the total binding free energy was decomposed into van der Waals, electrostatic, polar and nonpolar solvation contributions. Poisson–Boltzmann electrostatics were calculated using the APBS solver integrated within gmx_MMPBSA, with dielectric constants set to 80 for the solvent and 2 for the solute. The nonpolar solvation energy was estimated employing a SASA-based model. Final ΔG-bind values were computed by averaging over all snapshots, and the standard deviations were reported to reflect the variability across the sampled ensemble.

### 2.6. Allosteric Signaling and Mutational Analysis

Allosteric communication and mutational impact analyses were performed using the AlloSigMA algorithm [42], which applies a statistical mechanical model of protein energetics to predict the propagation of perturbations across residue networks. The three-dimensional structure of the target protein was uploaded in PDB format, and the ligand-binding residues were defined as the perturbation sites. The algorithm computes site-specific changes in free energy (Δh) upon virtual perturbation, thereby identifying energetically responsive residues and potential allosteric communication pathways. Per-residue free-energy profiles and Δh matrices were visualized to assess inter-chain coupling and cooperative effects. Predicted allosteric sites with significant energetic responses (|Δg| ≥ 1.0 kcal/mol) were further inspected for their spatial distribution relative to the catalytic and interface regions.

### 2.7. Structure Analysis and Representation

Protein three-dimensional structures were visualized and graphically represented using PyMol [43] and Protein Imager software (version 3.1.5) [44]. PyMOL was employed for structural manipulation, and visualization of dynamic residue variables and functional conservation. We used 3D Protein Imaging software to enhance graphical representations and produce publication-quality illustrations.

## 3. Results

### 3.1. Fungal Glutathione S-Transferase, Fhb7

*Fusarium* head blight resistance gene 7 (*Fhb7*) is a pivotal protein that has garnered attention for its role in combating Fusarium head blight (FHB), a devastating disease caused by *Fusarium* species, particularly *Fusarium graminearum*. A major virulence factor of this pathogen is its ability to produce DON, that disrupts protein synthesis in plants and causes significant agricultural and health concerns. The Fhb7 protein, originally identified in *Thinopyrum elongatum* and transferred to wheat through horizontal gene transfer, plays a central role in detoxifying DON and conferring resistance to *Fusarium* head blight [27]. Fhb7 encodes a glutathione S-transferase (GST)-like enzyme that detoxifies DON by catalyzing its conjugation with glutathione. This enzymatic reaction results in the formation of DON–glutathione conjugates, which are less toxic and more easily compartmentalized or metabolized by plant cells. The resulting DON–glutathione conjugates are sequestered into vacuoles or otherwise metabolized, preventing their interference with plant cellular processes [45].

A homology search of Fhb7 protein sequence using the BlastP algorithm against non-redundant NCBI protein data, showed that Fhb7 protein homologs are mainly present in fungal species, an observation which is compatible with a horizontal gene transfer phenomenon from fungi to plants (Figure S1). The Fhb7 sequence clustered within a well-supported clade composed predominantly of glutathione S-transferase (GST)-like proteins and hypothetical proteins from multiple fungal taxa, including species from the genera *Aspergillus*, *Trichoderma*, *Colletotrichum*, *Metarhizium*, and *Lasiodiplodia*. This fungal-dominated clade was clearly separated from other eukaryotic GST-like sequences, suggesting a distinct evolutionary origin. Within this major fungal cluster, Fhb7 grouped most closely with GST-like proteins from *Colletotrichum* species, including *C. tofieldiae* and *C. higginsianum*, which exhibit high sequence similarity and branch support, indicating a potential ancestral donor lineage. Other closely related sequences included putative GSTs from *Metarhizium* and *Trichoderma* spp., both of which are known to possess diverse detoxification enzymes, further supporting the hypothesis that Fhb7 was horizontally acquired from a fungus adapted to plant–pathogen interactions [27]. In contrast, plant-derived GST sequences were distributed in distant clades, demonstrating that Fhb7 does not share recent common ancestry with canonical plant GSTs. The presence of multiple functionally uncharacterized fungal proteins in proximity to Fhb7 also suggests that the GST lineage associated with DON detoxification is evolutionarily specialized and largely restricted to a subset of pathogenic and endophytic fungi (Figure S1).

**Figure 2:**
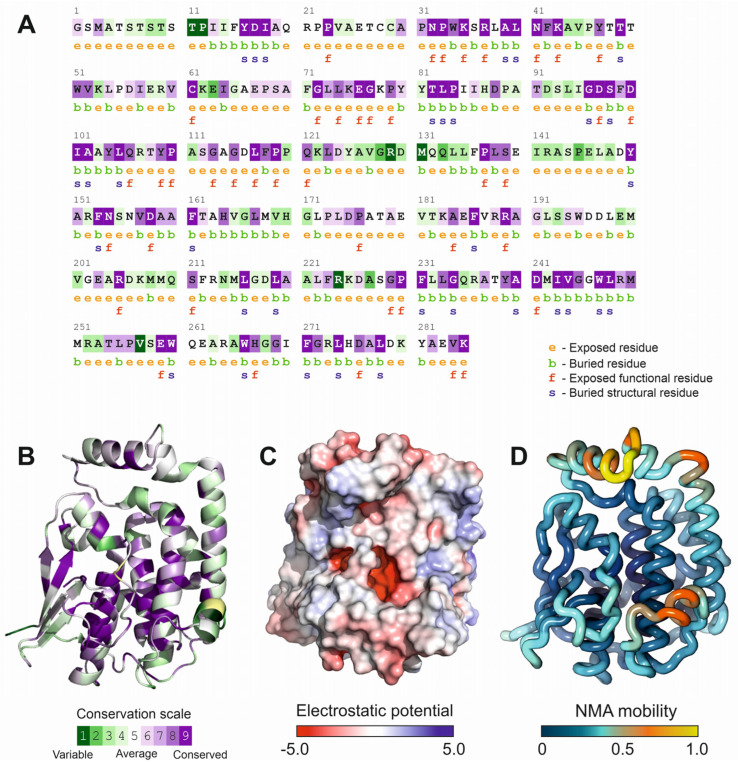
Sequence conservation and structural features of Fhb7 monomer from *Thinopyrum elongatum*. (**A**) Sequence conservation and residue characteristics across Fhb7 protein homologs, obtained by the ConSurf algorithm [31] applied to the top 100 hits and represented over *T. elongatum* protein sequence. (**B**) ConSurf conservation score results depicted over the canonical X-ray structure of Fhb7 from *T. elongatum*, that can be also translated to the sequence showed in panel (A)(PDB code: 7YOC). (**C**) Electrostatic potential over the surface of Fhb7 X-ray structure calculated by the APBS algorithm implemented in Pymol [46]. (**D**) Main chain mobility in Fhb7 structure calculated by normal mode analysis with the iMODs algorithm [34].

The availability of the crystal structure of Fhb7 protein (PDB code: 7YOC), allowed us to perform an estimation of the evolutionary conservation of the protein residues by exploiting the phylogenetic relationships among Fhb7 homologous sequences. Position-specific conservation scores were computed using the empirical Bayesian algorithm implemented in the ConSurf software [31], considering the top 100 homologous protein sequences. The results depicted in Figure 2A,B, showed the conservation scores represented by a colored gradient in the Fhb7 protein sequence and structure, respectively. Despite the presence of a canonical glutathione-transferase fold composed by two domains separated by a deep cleft, the family of Fhb7 homologs presents specific conserved regions. The N-terminal region residues from 23 to 43, located in the cofactor-binding domain and facing the interdomain cleft, are strongly conserved among Fhb7 homologs. Other conserved regions, mainly alpha helical in their structure, comprise residues from 74 to 120 and 229–249, being located at both sides of the interdomain cleft (Figure 2B). Interestingly, the cleft between both domains is partially covered by a flexible loop comprising residues 62 to 72, that shows a moderate conservation across Fhb7 homologs. Another flexible look between residues 123 and 143, involved in protein dimerization, is not conserved in the family members. The observation of conserved residues delimiting the interdomain region of Fhb7 protein prompted us to perform additional analysis to characterize this region. Calculation of continuous electrostatic potential the solution of the Poisson–Boltzman equation, and its representation over the protein surface (Figure 2C), showed that the interdomain cleft contains a surface patch with a negative electrostatic potential. This negatively charged patch is partially hidden to the solvent by the previously described flexible loop (amino acids from 62 to 72). Both the residue conservation and the presence of a negatively charged area suggested the possible involvement of this region of the protein in its catalytic activity.

**Figure 3:**
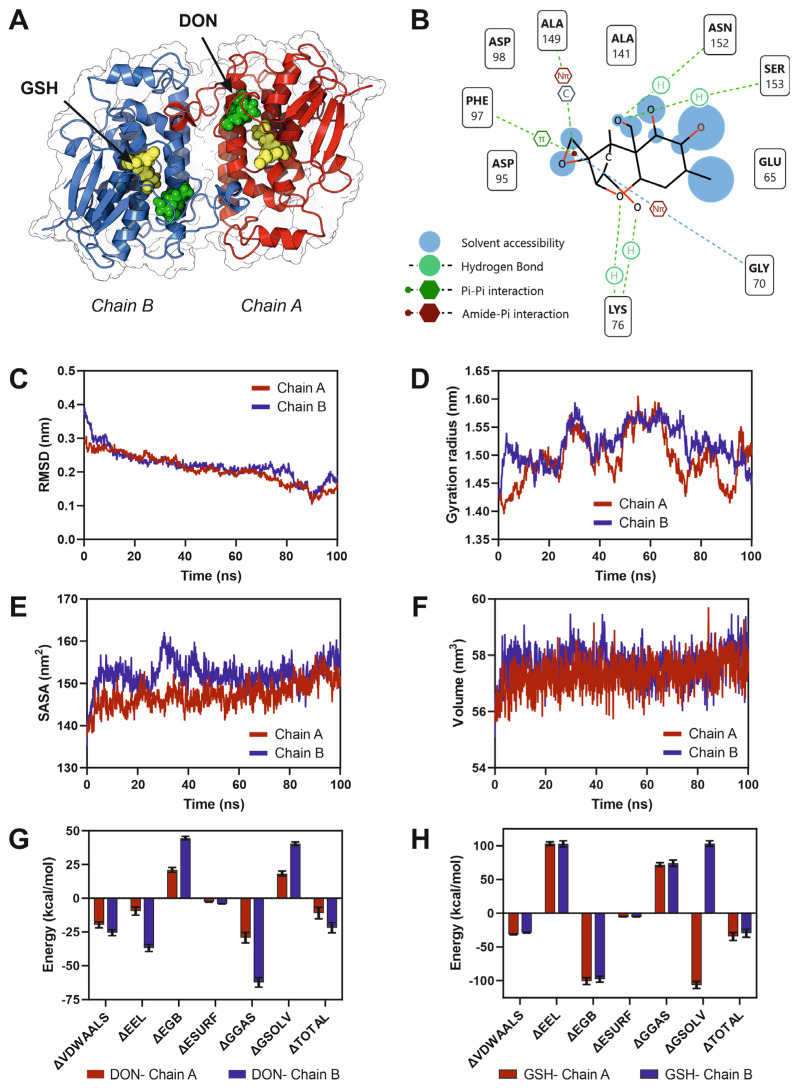
Structural basis and molecular dynamics of DON binding to the Fhb7 enzyme from *Thinopyrum elongatum*. (**A**) Overall structure of the Fhb7 homodimer (PDB: 7YOC) from *T. elongatum* showing the positioning of deoxynivalenol (DON, yellow spheres) and the natural cofactor glutathione (GSH, green spheres) within the active sites of chain A (red) and chain B (blue). (**B**) Two-dimensional schematic of the DON-binding pocket highlighting key interactions with Fhb7 active-site residues. Solvent-accessible regions are indicated in blue, illustrating partial pocket exposure. (**C**) Root-mean-square deviation (RMSD) of both monomer chains along the simulation. (**D**) Radius of gyration (Rg). (**E**) Solvent-accessible surface area (SASA). (**F**) Volume fluctuations in the enzyme dimer. (**G**) MM-PBSA binding free energy decomposition for DON in both enzyme monomers. (**H**) Comparative MM-PBSA analysis of GSH binding to both active sites. Numerical values and statistical parameters corresponding to panels (**G**,**H**) have been stored in Table S1.

To elucidate the molecular basis of deoxynivalenol (DON) recognition by the Fhb7 enzyme, we performed docking and 100 ns full-atom molecular dynamics (MD) simulations of the Fhb7 dimer in complex with DON and its natural cofactor, glutathione (GSH). The overall structure of the Fhb7 homodimer revealed two symmetric active sites, with each chain contributing to substrate recognition (Figure 3A). Docking analysis indicated that DON binds within a well-defined hydrophobic pocket adjacent to the GSH-binding site, stabilized by multiple hydrogen bonds and van der Waals contacts (Figure 3B). Key residues involved in DON interaction included Asp95, Asp98, Glu65, Lys76, Ser153, and Asn152, suggesting a cooperative network of hydrogen bonding and polar contacts critical for substrate positioning. MD simulations confirmed the stability of the DON–Fhb7 complexes, as indicated by low root-mean-square deviation (RMSD) values that stabilized around ~0.2 nm for both chains (Figure 3C). The radius of gyration (Rg) remained constant throughout the trajectory, indicating structural compactness of the enzyme during substrate binding (Figure 3D). Solvent-accessible surface area (SASA) analyses revealed slightly higher solvent exposure for chain B compared to chain A, suggesting subtle differences in local flexibility between subunits (Figure 3E). Similarly, the overall dimer volume remained stable across the simulations, reflecting conformational robustness of the complex (Figure 3F). Binding free energy calculations using the MMPBSA approach demonstrated strong and energetically favorable DON–Fhb7 interactions, with total binding energies of approximately −10 kcal·mol^−1^ for chain A and −21 kcal·mol^−1^ for chain B (Figure 3G). This notable asymmetry in binding affinities suggests that, despite the structural symmetry of the homodimer, the two catalytic pockets exhibit differential substrate stabilization. Such energetic disparity may indicate a potential allosteric behavior, where the binding of DON to one active site subtly influences the conformational dynamics and catalytic readiness of the opposite site. In contrast, GSH exhibited consistently stronger and nearly equivalent binding to both subunits (Figure 3H), consistent with its role as a cofactor tightly integrated into catalytic machinery. Collectively, these results suggest that DON is efficiently accommodated in the Fhb7 active site through a combination of polar and hydrophobic interactions, and that the enzyme dimer remains structurally stable upon substrate engagement. Moreover, the asymmetric binding energetics point to possible inter-subunit communication within the Fhb7 dimer, revealing an additional regulatory layer that could be exploited to engineer improved detoxification efficiency.

To study the potential allosteric events in DON catalytic centers in Fhb7 enzyme, we performed an energy coupling analysis using AlloSigMA algorithm. Our results revealed a pronounced energetic coupling between the two monomers of the *Fhb7* dimer, indicating a predicted allosteric relationship between both DON-binding sites (Figure S5). In the per-residue free energy profiles, several residues located within the DON-binding pockets of chains A and B exhibit negative ΔG values, reflecting a stabilizing response upon ligand perturbation. Notably, the allosteric response matrix demonstrates cross-chain communication patterns, where perturbations in the binding site of one subunit propagate through the dimer interface and influence corresponding residues in the partner chain (Figure S5). Together, these results support the existence of inter-subunit allosteric regulation within the *Fhb7* dimer, suggesting that DON binding to one active site may modulate the conformational dynamics and catalytic efficiency of the second site.

### 3.2. Bacterial Deoxynivalenol-3-Epimerase System: DepA and DepB

In the bacterial genus *Devosia*, the enzymatic detoxification of DON involves the oxidation of the C3 hydroxyl group, forming a 3-keto intermediate [47]. Subsequently, this intermediate undergoes stereospecific reduction to yield 3-epi-DON, which exhibits significantly reduced toxicity compared to its parent molecule. DepA, encoded by the depA gene, is a quinone-dependent oxidase that catalyzes the first step in the detoxification pathway [48]. Unlike NAD+- or NADP+-dependent enzymes, DepA uses pyrroloquinoline quinone (PQQ) as a redox-active cofactor. PQQ enables efficient electron transfer during the oxidation of the hydroxyl group at the C3 position of DON, resulting in the formation of a 3-keto intermediate [49]. The 3-keto intermediate generated by DepA is acted upon by DepB, encoded by the *depB* gene. DepB reduces the 3-keto group to a hydroxyl group, specifically flipping its stereochemistry to form 3-epi-DON. This reduction step requires a reductant, likely a nicotinamide cofactor such as NADH or NADPH, to donate electrons for the reduction process. The activity of DepB ensures the completion of the detoxification pathway, converting a highly toxic compound (DON) into a less harmful epimeric form (3-epi-DON). The stereochemical precision of DepB highlights its evolutionary adaptation to detoxify trichothecenes efficiently [50].

The phylogenetic reconstruction of the DepA enzyme from *Devosia* reveals a well-defined clustering of sequences within the Alphaproteobacteria clade, highlighting a strong evolutionary conservation of this enzyme among members of the genus *Devosia* (Figure S2). The DepA sequence used as the reference groups tightly with other *Devosia* homologs, including those from *D. yakushimensis*, *D. faecipullorum*, and unclassified *Devosia* species, suggesting the existence of a conserved catalytic mechanism for DON detoxification within this lineage. Outside the *Devosia* cluster, more distant homologs were identified in related *Sphingomonadales* genera, such as *Sphingomonas*, *Ketogulonicigenium*, and *Pseudogluconobacter*, indicating possible functional conservation across taxa but with increasing sequence divergence. Interestingly, the tree topology also reveals several deeply branching groups within Acidobacteriota and Gammaproteobacteria, which form outgroups and likely represent ancestral diversification events of oxidoreductases with related structural domains but potentially distinct substrate specificities. The clustering pattern supports the hypothesis that the DON-degrading DepA enzyme evolved within Alphaproteobacteria, with horizontal gene transfer playing a minor role, as suggested by the strong genus-level conservation.

**Figure 4:**
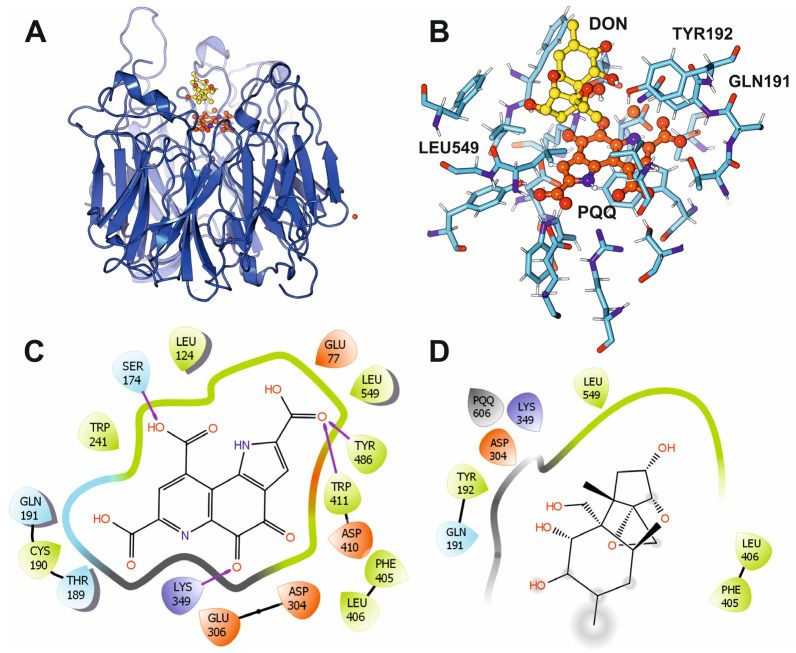
Structural insights into the active site architecture and substrate–cofactor interactions of the DepA enzyme from *Devosia* sp. involved in DON degradation. (**A**) Side view of the structure of DepA showing the characteristic β-propeller fold and the central catalytic pocket where the substrate DON (yellow) and the PQQ cofactor (orange) are bound (PDB ID: 7WMD). (**B**) Close-up view of the active site, illustrating the spatial organization of DON, PQQ, and key catalytic residues (Tyr192, Gln191, Leu549) that stabilize substrate positioning and facilitate electron transfer. (**C**) Two-dimensional interaction map of PQQ with surrounding amino acid residues, highlighting hydrogen bonds (purple arrows), hydrophobic contacts, and electrostatic interactions that secure the cofactor within the catalytic cavity (PDB code: 7WMK). (**D**) Detailed view of the DON-binding pocket after flexible substrate docking, showing hydrophobic contacts formed by Phe405, Leu406, and Leu549, together with polar interactions mediated by Gln191 and Tyr192, which precisely orient DON for stereospecific oxidation. The purple- and brown-marked amino acids correspond to residues involved in electrostatic interactions, the green amino acids are involved in hydrophobic interactions, and the blue-marked residues are establishing polar interactions with the cofactor or substrate.

The crystal structure of DepA from *Devosia* sp. was previously determined in the APO (PDB ID: 7WMD) and PQQ-bound (PDB ID: 7WMK) forms. The overall DepA structure comprises an eight-bladed β-propeller fold, typical of Type I PQQ-dependent alcohol dehydrogenases, with a central catalytic pocket that should accommodate both the PQQ cofactor and the DON substrate. Notably, four loops form a mobile “lid” over the active site; three are stabilized by PQQ, and a fourth one appears to be implicated in substrate capture and selectivity. Flexible blind docking of DON on the PQQ-bound protein structure into the catalytic cavity highlighted the positioning of PQQ relative to DON and its surrounding network of hydrogen bonds and hydrophobic contacts (Figure 4A). Key residues, including Gln191, Tyr192 and Leu549, are strategically located to stabilize substrate binding and properly orient DON for catalysis. PQQ is deeply buried within the active site and anchored through extensive interactions with surrounding amino acids, ensuring its correct positioning to mediate the two-electron transfer required for DON oxidation. The alignment between PQQ’s reactive carbonyl group and the C3-hydroxyl of DON indicates the mechanistic basis for the conversion of DON into 3-keto-DON, the first intermediate in the epimerization pathway (Figure 4B). Moreover, Trp241, Asp304, Lys349, Phe405, Asp410 and Tyr486 form stabilizing hydrogen bonds and hydrophobic contacts that secure the cofactor within the catalytic site. The interactions involving Lys349 and Asp304 appear to maintain local electrostatic balance, potentially facilitating charge redistribution during the oxidation step (Figure 4C). In the DON-binding pocket, residues Phe405, Leu406, and Leu549 form a hydrophobic cradle, while Gln191 and Tyr192 establish polar contacts that align the substrate within the active site. This precise arrangement likely restricts substrate mobility, ensuring that the C3-hydroxyl group of DON is optimally positioned for hydride abstraction and electron transfer to PQQ (Figure 4D). Recent biochemical work further shows DepA accepts several trichothecene variants (e.g., 15-acetyl-DON), underscoring a binding mode tolerant to C15 substitutions while preserving the C3 redox geometry, behavior explainable by the spacious, loop-defined portal observed crystallographically [47].

The DepB crystal structure (PDB code: 8HWN) captures the enzyme in an apo-like state with bound ions but no nicotinamide cofactor. Fold assignment and annotations identify DepB as an aldo-keto reductase (AKR), which canonically adopts a (β/α)_8_-barrel with a conserved catalytic tetrad (Tyr–Lys–Asp–His) at the mouth of the barrel and a NADPH-binding site shaped to recognize the 2′-phosphate. Although the cofactor is absent in 8HWN, the AKR architecture predicts an ordered bi–bi mechanism in which NADPH binds first; Tyr acts as the general acid/base; and hydride is delivered to the re-face/si-face of the 3-keto-DON carbonyl to enforce inversion at C3, yielding 3-epi-DON. Biochemistry shows strong NADPH preference and robust activity over neutral pH, aligning with AKR family behavior [25].

The phylogenetic analysis of the DepB enzyme, constructed from a BlastP-derived multiple sequence alignment of homologous aldo/keto reductases (AKRs), revealed a distinct evolutionary clustering pattern (Figure S3). DepB from *Devosia* sp. grouped within a well-supported clade together with close homologs from other *Devosia* strains and the related genus *Paradevosia*, indicating a recent divergence within this lineage. This cluster was clearly separated from more distantly related AKR family members from *Rhizobium*, *Plantimonas*, and other Alphaproteobacteria, which formed several independent subclades. The topology suggests that DepB and its closest homologs likely share a conserved catalytic mechanism for DON epimerization, while the divergence from other AKR clades indicates functional specialization within *Devosia* and its relatives. The tight grouping of *Devosia* sequences, supported by high bootstrap values, is consistent with the enzyme’s niche adaptation for DON biodegradation, whereas the broader AKR diversity highlights potential structural variability and substrate promiscuity across bacterial lineages.

**Figure 5:**
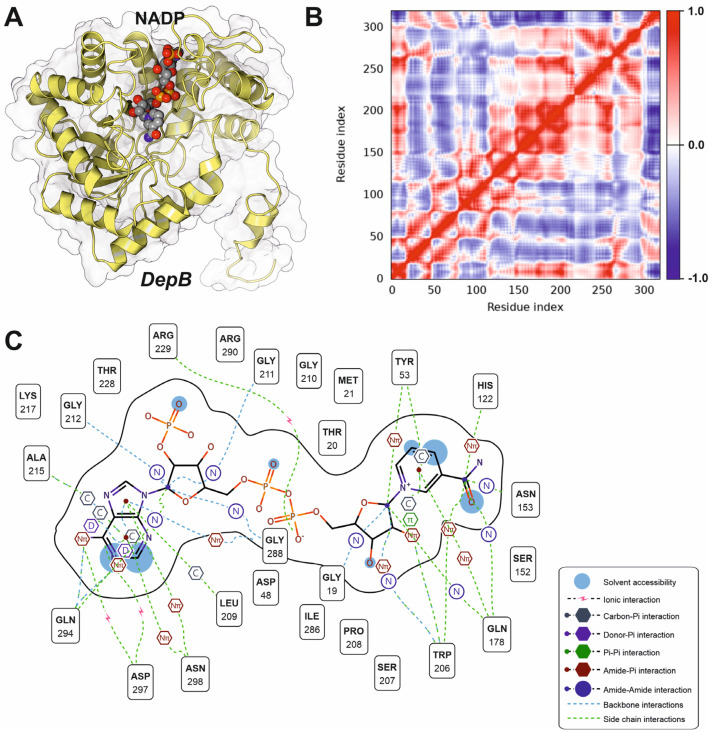
Structural characterization of the NADP-binding site and dynamic coupling in DepB. (**A**) Overall structure of DepB highlighting the NADP-binding pocket. The enzyme is shown in ribbon representation (yellow) with the molecular surface displayed in transparent gray, while the bound NADP molecule is depicted as spheres (carbon, gray; oxygen, red; nitrogen, blue; phosphate, orange). The cofactor is positioned within a deep hydrophobic cleft at the interface of several α-helices and β-strands. (**B**) Covariance matrix obtained from normal mode analysis using iMODS, showing residue–residue dynamic correlations within DepB. Positive correlations (red) indicate coordinated motions, particularly within the NADP-binding region, whereas anti-correlated motions (blue) correspond to distal structural domains, suggesting functional compartmentalization of the enzyme. (**C**) Detailed two-dimensional interaction map of the amino acid residues involved in NADP binding. Solvent accessibility, backbone and side-chain contacts, and various π-interactions are indicated according to the legend.

Docking analysis of the NADP binding site in DepB revealed the architecture of the NADP-binding pocket and the molecular interactions stabilizing cofactor binding (Figure 5A,C). Hydrogen bonding and polar contacts dominate NADP interaction, with critical contributions from residues Gly211, Gly212, Arg229, and Gly288. Additional stabilizing interactions are mediated by Ser152, Asn153, Gln178, Ser207, Lys217, and Arg290, which form a hydrogen-bonding network that anchors the cofactor in an optimal orientation for catalysis. Several residues, including Tyr53 and Trp206, participate in π–π and donor–π stacking interactions, providing further stabilization of the adenine ring system. The dynamic behavior of the NADP-binding region was analyzed by normal mode calculations using iMODS. The analysis revealed strong positive correlations (red regions) among residues forming the NADP-binding pocket, indicating a high degree of coordinated motion within the catalytic site (Figure 5B). In contrast, distal structural segments displayed weaker or anti-correlated motions (blue regions), suggesting functional partitioning between the active site and peripheral regions of the enzyme. These results indicate that cofactor binding induces a structurally constrained yet dynamically coordinated environment around the catalytic core, potentially facilitating efficient hydride transfer to keto-DON. However, docking algorithms were not successful in the determination of the substrate pocket for keto-DON, suggesting a transient interaction phenomenon or a possible induced folding facilitated by the substrate.

### 3.3. Plant Deoxynivalenol Glyoxalase, SPG Glyoxalase

The glyoxalase system, comprising glyoxalase I (GLO1) and glyoxalase II (GLO2), primarily detoxifies methylglyoxal (MG), a cytotoxic byproduct of glycolysis [51]. Recent evidence described a specialized glyoxalase I enzyme in *Gossypium hirsutum* (SPG glyoxalase) able to detoxify trichothecenes, including DON [52]. The SPG enzyme exhibits unique catalytic properties that enable it to detoxify DON. Structural studies by X-ray crystallography have revealed that SPG has a distinct active site configuration, allowing it to specifically interact with DON molecules [28].

Since many plant species are subjected to the selective pressure of *Fusarium* sp. infections and its mycotoxins, we investigated if the SPG enzyme is conserved among other plants. A homology search of SPG protein sequence using the BlastP algorithm showed that SPG protein homologs are mainly present in plant species. Moreover, the phylogenetic tree, constructed from the top 250 homologues of *Gossypium* SPG protein, provided further insights into the evolutionary history and functional diversification of this protein family (Figure S4). The phylogenetic tree showed that SPG protein sequences from *Gossypium* species (*G. australe*, *G. harknessii*, *G. stocksii* and *G. hirsutum*) formed a separate and central clade, suggesting the existence of potential evolutionary pressures imposed by the coevolution of plants together with DON-producing *Fusarium* species. Monocots species such as *Musa acuminata* (banana) and *Zingiber officinale* (ginger) exhibit divergence in glyoxalase sequences, reflecting adaptation to high metabolic demands in tropical environments. Fabaceae (*Glycine max*, *Pisum sativum*) form a distinct clade reflecting their metabolic specialization in processes such as symbiotic nitrogen fixation. Brassicaceae (*Arabidopsis thaliana*, *Brassica napus*), exhibit robust glyoxalase diversification, likely to reflect their adaptation to oxidative stress and defense against pathogens. Glyoxalase proteins in agriculturally significant species (*Triticum aestivum*, *Oryza sativa*) showed convergent evolution with *Gossypium*, reflecting adaptations to fungal pathogens in cultivated systems. The central clade location of SPG glyoxalase proteins in *Gossypium* suggested that this gene underwent gene duplication, with specific paralogs evolving enhanced activity against DON. These duplications may have been driven by selective pressure from recurrent exposure to *Fusarium* pathogens. The phylogenetic analysis suggested the putative presence of SPG homologs in other neighbor plant species that could be selected for DON degradation by the environmental pressure exerted from *Fusarium* infections.

**Figure 6:**
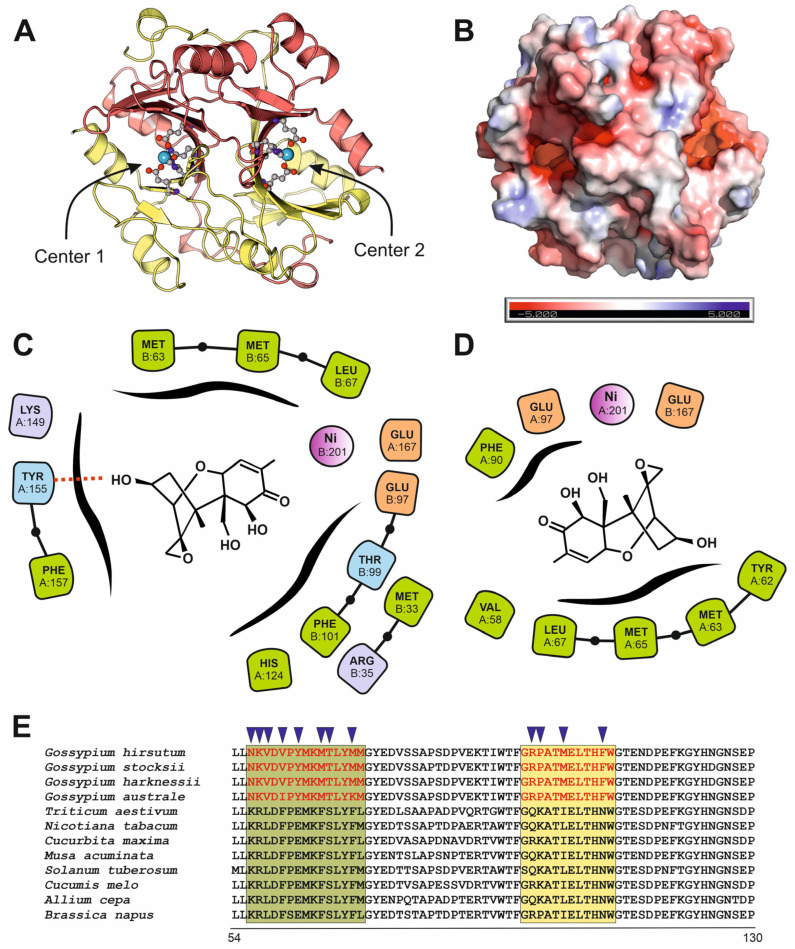
Structural and functional characteristics of SPG glyoxalase from *Gossipyum hirsutum*. (**A**) Ribbon representation of the SPG dimer as determined by X-ray crystallography experiments (PDB code: 7VQ6), showing the intricated monomer interconnection and the presence of two Nickel centers. (**B**) Electrostatic potential distribution over the surface of the SPG dimer. (**C**) Contact diagram of the amino acids in the putative DON binding close to Ni Center 1 and located at less than 3.5 Å, as determined by blind docking with CB-Dock2 [35]. (**D**) Contact diagram of the amino acids in the putative DON binding close to Ni Center 2 and located at less than 3.5 Å, as determined by blind docking. The purple- and brown-marked amino acids correspond to residues involved in electrostatic interactions, the green amino acids are involved in hydrophobic interactions, and the blue-marked residues are establishing polar interactions with the substrate. (**E**) Sequence alignment of amino acids 54–130 in SPG sequence, showing the difference between the species belonging to the *Gossypium* genus, and other plant species that could also be infected by *Fusarium*. Colored squares depict the two analyzed motifs, whereas triangles locate the amino acids that are different in *Gossypium*, compared to other plants that could also be infected by *Fusarium*.

Based on the crystal structure of SPG enzyme from *G. hirsutum* (PDB code: 7VQ6), we performed a detailed analysis to characterize the residues involved in its enzymatic activity. The enzyme is a dimer that contains two Nickel centers, conserved in a wide family of glyoxalases (Figure 6A). Both centers are located within surface cavities within negative electrostatic patches (Figure 6B). The original report of the SPG crystal structure by Hu and coworkers [28], postulates that the catalytic center of the *Gossypium* enzyme has evolved to accommodate DON despite its original function as glutathione binder. This evolutive path has inferred from the striking differences in the residues present in the catalytic center of the *Gossypium* enzyme in comparison to similar enzymes from bacteria and animals. However, the symmetric dimer also suggested the presence of two potential active centers, located close to both Nickel atoms. We performed a blind docking with surface pocket detection to determine the putative positions of surface pockets able to bind DON. In the ranked list of docking solutions, we confirm the previously described catalytic center, but we also determined another putative catalytic pocket located in the negative patch close to the second Nickel atom. The estimated binding energies for DON-SPG complexes are similar for both catalytic sites, being −7.67 and −7.31 kcal/mol, respectively. Analyzing the protein residues involved in interactions with DON and located closer than 3.5 Å from the mycotoxin in the docking solutions, we determined that Glu167 and Glu97, residues that are involved in the coordination of the Nickel atoms, are near to the mycotoxin in the binding predictions. Additionally, Met63, Met65 and Leu67 also appeared as interacting residues in both docking solutions (Figure 6C,D). Expanding the analysis to the residues located up- and downstream, we identified a 14 amino acid signature that is unique in the SPG proteins protein within *Gossypium* genus (Figure 6E). The peptide from amino acids 56 to 69 in *Gossypium* has the sequence NKVDVPYMKMTLYM, whereas in other plant species also infected by Fusarium has the canonical sequence KRLDFPEMKFSLYF. This difference results in decrease in the polarity, measured as the grand average of hydropathicity (GRAVY) index of this region (−0.13 for *Gossypium* proteins and −0.36 for other species) and a decrease in the aromaticity (0.14 for *Gossypium* and 0.28 for other species). The subsequent decrease in the polarity of this region of the *Gossypium* protein induces a difference in the folding compared to the glyoxalases from other plants, that results in a more exposed area and an increment in the dimensions and volume of the putative substrate-binding pocket. Another *Gossypium*-specific signature is located downstream, comprising residues 92 to 101. In *Gossypium* it has the sequence RPATMELTHF, whereas in other plants it has the canonical sequence QKATIELTHN (Figure 6E). The analysis of physicochemical properties of this second motif is consistent with a decrease in the polarity in *Gossypium* enzymes (GRAVY index of −0.39 for *Gossypium* enzymes and −0.89 for other species). To determine additional physicochemical parameters, we performed a calculation of the electrostatic properties of the putative binding pockets of the SPG protein and a glyoxalase from *T. aestivum* by solving the Poisson–Boltzmann equation. The results clearly showed that the abundance of polar residues in the *Gossypium* protein around the putative catalytic pocket resulted in an increase in the negative potential of this region, compared to the same region in the glyoxalase of *T. aestivum* (Figure S6).

We also studied the coevolutionary relationships among residue pairs in glyoxalase multiple sequence alignments, using the mutual Information (MI) algorithm included in MISTIC2 software [32]. MI measures the statistical dependence between two positions in the sequence alignments. The MISTIC2 coevolutionary analysis of the SPG protein revealed distinct patterns of residue conservation and inter-residue communication (Figure S7). Several highly conserved regions, notably around positions 45–74, 99–125, and 158–170, were detected, suggesting their potential involvement in maintaining the protein’s structural integrity or catalytic activity. The analysis of high-confidence mutual information (MI) connections, with MI scores exceeding 5.0, indicating strong evolutionary coupling between distant residues. Prominent coevolving clusters were detected in the N-terminal segment (positions 33–50) and distal regions near 138–159, suggesting long-range intramolecular interactions critical for the stabilization of the protein fold. Additional MI links among central residues (50–60, 95–101) suggest the existence of an internal communication network likely contributing to conformational dynamics. Collectively, these results support the presence of a coordinated network of evolutionarily constrained residues, implying their central role in SPG structural stability and functional regulation. Notably, Met96 displayed a highly significant coevolutionary relationship with residue Met29 (MI Score: 43.82), suggesting an adaptative role in maintaining interactions with a more conserved structural or catalytic site. Similarly, residues Arg175 and Val178 showed a strong coevolutionary connection (MI Score: 45.90), with both residues displaying moderate to high conservation scores (2.39 and 2.47, respectively), implying a local stabilizing role in the protein’s structural framework. Additional coevolution pairs include Arg175-Ile177 (MI Score: 42.93), Ile177-Val178 (MI score: 35.48), and Gly173-Arg175 (MI Score: 26.79) belonging to a potential functional motif or domain interface (Figure S7). The overall conservation patterns indicate that these residues contribute to the structural integrity and functional dynamics of the SPG protein. These findings provide key insights into the coevolutionary constraints shaping the SPG protein’s function and offer candidate sites for further structural and mutational studies to validate their biological roles.

## 4. Discussion

Our integrative analysis combining phylogenetics, structural modeling, docking, and molecular dynamics (MD) simulations reveals new insights into the evolutionary origin and catalytic mechanism of the Fhb7 enzyme in *T. elongatum*, a key factor in DON detoxification and *Fusarium* head blight resistance. Phylogenetic analysis places Fhb7 within a distinct fungal-dominated clade, showing highest similarity to GST-like proteins from *Colletotrichum* species. These findings support a horizontal gene transfer (HGT) event from fungi to the *Thinopyrum* lineage, consistent with an endophytic rather than canonical plant GST origin [53]. Moreover, our broader taxonomic survey extends these observations by identifying additional potential donor lineages, including detoxification-specialized GSTs from *Metarhizium* and *Trichoderma*. Some *Metarhizium* species have also been described by their potential for gene transfer to plants, modulating the resistance to different pathogens [54]. The restricted distribution of homologous sequences suggests a specialized evolutionary adaptation for DON detoxification, reinforcing the idea that Fhb7 acquisition conferred a selective advantage to the host genome under *Fusarium*-driven environmental pressure [55].

Residue conservation mapping highlights three regions of high evolutionary constraint surrounding the interdomain cleft, notably residues 23–43 and 74–120, which correspond to the GSH-binding domain and structural helices adjacent to the catalytic pocket. The presence of a negatively charged electrostatic patch within this cleft (Figure 2C), partially shielded by a flexible loop (residues 62–72), suggests its role in substrate recognition and positioning. Importantly, the lack of conservation in dimerization-associated loops (residues 123–143) points to a functionally divergent interface, potentially contributing to the observed binding asymmetry between Fhb7 subunits. Docking and MD simulations revealed a well-defined DON-binding pocket stabilized by polar contacts involving Glu65, Asp95 and Asp98, and by additional hydrogen bonds with Lys76, Asn152 and Ser153, alongside hydrophobic interactions. Additionally, the striking binding free energy disparity between chains A and B (−10 kcal·mol^−1^ vs. −21 kcal·mol^−1^) suggests an asymmetric catalytic environment within the homodimer pointing to a potential allosteric behavior of the enzyme. Given the structural symmetry of the dimer, this asymmetry likely reflects subtle inter-subunit conformational differences or long-range allosteric effects, as supported by the increased solvent exposure and flexibility observed in chain B [56]. Such dynamics may represent a mechanistic tuning strategy, where differential DON stabilization allows the enzyme to balance catalytic efficiency with cofactor recycling. Similar allosteric behavior in glutathione transferases has been described in higher eukaryotes, including humans [57].

Fhb7 employs a glutathione-dependent mechanism targeting the epoxide group of DON, consistent with previous biochemical evidence. Our data expand upon this model by revealing that residues forming the electrostatically negative interdomain cleft, together with the partially shielding flexible loop, create a structurally gated environment optimizing substrate orientation. Importantly, the presence of highly conserved acidic residues (Asp95, Asp98) within the catalytic pocket provides a plausible explanation for the observed substrate specificity, distinguishing Fhb7 from canonical plant GSTs, which typically act on broader xenobiotic profiles [58,59]. Our findings suggest that Fhb7 functions as a structurally optimized, dynamically regulated GST, uniquely adapted for DON detoxification. The asymmetric stabilization of DON within the dimer highlights a previously unreported layer of potential allosteric regulation, opening avenues for rational engineering. By targeting conserved residues within the catalytic cleft or modifying the flexible loop that modulates pocket accessibility, it may be possible to enhance enzyme efficiency or broaden substrate range [60].

The enzymatic detoxification of deoxynivalenol (DON) by *Devosia* species represents one of the most efficient bacterial strategies for mitigating trichothecene toxicity. In this pathway, DepA catalyzes the initial oxidation of the C3-hydroxyl group of DON, producing a 3-keto intermediate, which is subsequently reduced by DepB to yield 3-epi-DON. DepA is a rare example of a pyrroloquinoline quinone (PQQ)-dependent oxidoreductase, distinguished from classical NAD(P)H-dependent alcohol dehydrogenases by its utilization of a PQQ cofactor to mediate electron transfer. Structural analyses of the PQQ-bound crystal structure (PDB ID: 7WMK) revealed an eight-bladed β-propeller fold typical of Type I PQQ-dependent enzymes, with the catalytic site buried deep within the central cavity. Docking simulations confirmed that PQQ is tightly coordinated by an extensive hydrogen-bonding network that facilitates optimal positioning for hydride abstraction from the C3-hydroxyl of DON. The hydrophobic cradle formed by Phe405, Leu406, and Leu549 stabilizes the substrate, while polar contacts from Gln191 and Tyr192 precisely align DON for catalysis. This arrangement explains the enzyme’s high stereospecificity and supports its ability to accommodate various DON derivatives, including 15-acetyl-DON, without disrupting catalytic geometry [61]. In contrast, DepB is an aldo-keto reductase (AKR) that catalyzes the stereospecific reduction in the DepA-generated 3-keto intermediate, completing the formation of 3-epi-DON [50]. The crystal structure (PDB ID: 8HWN) shows the canonical (β/α)_8_-barrel fold and a conserved catalytic tetrad (Tyr–Lys–Asp–His) typical of AKR family enzymes. Docking analyses of the NADP-binding site revealed a highly coordinated hydrogen-bonding network involving Gly 211, Gly212, Arg229 and Gly288. These residues position NADPH in a geometry optimized for hydride delivery to the 3-keto carbon, enforcing inversion of stereochemistry at C3 [62]. Interestingly, our covariance analysis of DepB dynamics showed strong positive correlations among residues within the NADP-binding site, indicating a structurally constrained yet highly coordinated catalytic environment. This dynamic coupling likely facilitates efficient cofactor-assisted electron transfer while maintaining substrate specificity [25].

The phylogenetic analyses of DepA and DepB further revealed tight clustering of homologs within *Devosia*, suggesting that these enzymes coevolved as part of a specialized DON detoxification module. Distant homologs in related Alphaproteobacteria, such as *Paradevosia* and *Sphingomonas*, likely retain ancestral oxidoreductase scaffolds but exhibit divergent substrate preferences [63]. The strong conservation of active-site residues within the *Devosia* clade supports the hypothesis that DON detoxification arose under specific ecological pressures, favoring efficient epimerization pathways in bacterial species inhabiting cereal-associated environments [64]. Together, these findings provide a detailed structural and mechanistic framework for understanding DON epimerization in *Devosia*. DepA’s PQQ-mediated oxidation and DepB’s AKR-driven reduction operate in a highly coordinated, sequential manner, ensuring high stereospecificity and efficiency. Insights from these analyses not only elucidate the molecular basis of DON detoxification but may also guide future protein engineering efforts to enhance catalytic activity or expand substrate specificity for biotechnological applications in food safety.

In response to the persistent toxic challenge imposed by *Fusarium* pathogens and their trichothecene mycotoxins, such as deoxynivalenol (DON), plants including *Gossypium* species have evolved to produce a variety of biochemical and molecular defense systems aimed at mitigating toxin-induced damage. Among these, the specific protein glyoxalase-like enzyme (SPG glyoxalase) has emerged as a key component with the ability to metabolize and neutralize DON. The evolution of SPG highlights the intense selective pressure exerted by *Fusarium* on the *Gossypium* genome, representing a paradigmatic example of host–pathogen coevolution. This adaptive process likely stems from a long-standing evolutionary arms race in which recurrent episodes of *Fusarium* infection and toxin exposure have continually reshaped the plant’s metabolic repertoire. The presence of DON as a persistent environmental stressor may have provided the necessary selection pressure for the recruitment and diversification of ancestral glyoxalase-like enzymes toward novel detoxification functions [55]. Our phylogenetic and structural analyses reinforce the hypothesis of functional divergence within the glyoxalase enzyme family, suggesting that gene duplication followed by adaptive mutations in the active site enabled the emergence of DON-degrading activity. Under DON exposure, *Gossypium* plants experience heightened oxidative stress and disturbances in cellular homeostasis, leading to the accumulation of reactive aldehydes such as methylglyoxal. The simultaneous need to detoxify both methylglyoxal and DON may have imposed a dual selective constraint on glyoxalase genes, thereby accelerating their neofunctionalization.

The discovery of two Ni-centered sites in the *Gossypium* SPG dimer is consistent with the well-established metal dependence of type I glyoxalase enzymes (GLYI) and helps rationalize SPG’s expanded substrate scope toward trichothecenes. Recent surveys show that GLYI can be Zn^2+^- or Ni^2+^-activated, with plants capable of encoding both metal classes; notably, *Arabidopsis thaliana* harbors distinct Ni^2+^- and Zn^2+^-dependent isoforms, highlighting metal tuning as a key evolutionary mechanism driving catalytic diversification [65]. The presence of two Ni-associated pockets in the SPG dimer raises the possibility of cooperative or partially redundant catalytic sites, a scenario compatible with GLYI’s dimeric scaffolding in other systems and with metal-center-guided substrate positioning [66].

SPG thus exemplifies an evolutionary innovation driven by biochemical necessity and environmental challenge, a newly specialized enzyme that integrates detoxification of xenobiotic compounds into the broader stress-response network of the plant [67]. SPG represents a prime example of how such evolutionary forces can result in the emergence of a novel enzyme with a specialized role in host defense. Comparative genomic provided further insight into the evolution of SPG. Sequence data suggest that SPG shares structural similarities with glyoxalases but has distinct active site adaptations for DON recognition and degradation [28]. Elevated expression of SPG in *Gossypium* upon *Fusarium* infection and DON exposure further underscores its importance as a key defensive adaptation. The interaction between *Fusarium* and *Gossypium* exemplifies a coevolutionary dynamic where the pathogen’s production of DON pressures the host to develop innovative detoxification strategies [68].

The comparative analysis of DON biodegradation systems across bacteria, fungi, and plants highlights that equivalent detoxification outcomes have arisen through distinct evolutionary pathways shaped by strong selective pressures imposed by *Fusarium*-derived mycotoxins. In bacteria, the DepA/DepB-mediated epimerization system is highly conserved within *Devosia* and closely related Alphaproteobacteria, indicating a predominant mode of vertical inheritance that preserves the catalytic efficiency of PQQ-dependent oxidation followed by NADPH-driven stereospecific reduction [25,26]. However, the detection of functionally analogous enzymes in taxonomically distant genera, such as *Pelagibacterium*, *Nocardioides*, and *Sphingomonas*, suggests episodes of functional convergence, likely reflecting repeated selective pressures in DON-rich environments [69]. This convergence is supported by structural similarities in active-site architecture despite divergent sequence backgrounds, implying that detoxification capability emerged multiple times via recruitment of pre-existing oxidoreductase scaffolds optimized for DON recognition and catalysis [70].

In contrast, plants have evolved detoxification solutions through fundamentally different genomic routes. The Fhb7 gene in *Thinopyrum elongatum* represents a striking example of horizontal gene transfer (HGT), as revealed by our phylogenetic reconstruction, which places Fhb7 within a fungal glutathione S-transferase (GST) clade, with closest homology to *Epichloë*-like endophytes [27]. This cross-kingdom acquisition provided an immediate adaptive advantage, enabling efficient DON detoxification via glutathione conjugation and epoxide ring opening—mechanisms absent from canonical plant GSTs. Such HGT events have been increasingly recognized as significant evolutionary drivers, equipping plants with entirely new metabolic pathways that expand chemical defense repertoires under pathogen-driven selection [71]. Following its acquisition, Fhb7 appears to have undergone rapid neofunctionalization, with structural adaptations such as a negatively charged interdomain cleft, conserved GSH-binding motifs, and dynamic gating loops that optimize DON recognition and catalysis, as supported by our docking and MD simulation results.

A third and distinct trajectory is represented by the SPG glyoxalases of *Gossypium* species, which evolved not through HGT but via gene duplication and adaptive diversification within canonical plant glyoxalase families [25]. Our structural and phylogenetic analyses reveal that SPG paralogs in *Gossypium* underwent selective remodeling of their substrate-binding pockets, increasing local polarity and altering electrostatic potential to accommodate DON while preserving their ancestral role in methylglyoxal detoxification. These modifications are consistent with signatures of positive selection, suggesting that SPG neofunctionalization was driven by recurrent exposure to *Fusarium* toxins and the associated ecological arms race. Notably, while SPG homologs are broadly distributed across plants, the enhanced DON-detoxifying capacity of the *Gossypium* clade appears to result from lineage-specific fine-tuning rather than acquisition of entirely novel enzymatic systems. Nevertheless, future work should consider more specific methods such as codon-level analysis to test for positive selection and adaptive evolution.

## 5. Conclusions

In conclusion, our comparative analyses reveal that DON detoxification has emerged through multiple, evolutionarily distinct solutions shaped by intense selective pressures imposed by *Fusarium* pathogens. While the DepA/DepB epimerization pathway in *Devosia* represents a highly conserved, vertically inherited bacterial strategy, the Fhb7-mediated GST conjugation in *T. elongatum* exemplifies adaptive innovation via horizontal gene transfer, and the SPG glyoxalases in *Gossypium* highlight neofunctionalization within plant enzyme families. Despite their divergent origins, these systems converge on a shared outcome: efficient recognition and structural transformation of DON into less toxic derivatives. The structural insights provided by our docking, molecular dynamics simulations, and evolutionary analyses illuminate how distinct catalytic architectures have been repeatedly optimized for substrate specificity and enzymatic efficiency. Beyond deepening our understanding of DON biodegradation, these findings provide a mechanistic framework for the rational engineering of enzymes with enhanced detoxification capacity, offering promising strategies that could be used for developing Fusarium-resistant crops and improving food safety.

## Data Availability

The raw data supporting the conclusions of this article will be made available by the authors on request.

## Supplementary Materials

The following supporting information can be downloaded at: https://www.mdpi.com/article/10.3390/microorganisms13102384/s1, Figure S1: Phylogenetic analysis of the Fhb7 protein from *Thinopyrum elongatum* and homologous sequences from diverse fungal and plant species. The phylogenetic tree was generated using amino acid sequences identified by BlastP searches against the NCBI nr database, with the Fhb7 sequence used as the query. Multiple sequence alignment was performed using Clustal Omega, and the tree was inferred using the Maximum Likelihood method; Figure S2: Phylogenetic analysis of the DepA protein from *Devosia* sp. and homologous sequences from diverse fungal and plant species. The phylogenetic tree was generated using amino acid sequences identified by BlastP searches against the NCBI nr database, with the DepA sequence used as the query. Multiple sequence alignment was performed using Clustal Omega, and the tree was inferred using the Maximum Likelihood method; Figure S3: Phylogenetic analysis of the DepB protein from *Devosia* sp. and homologous sequences from diverse fungal and plant species. The phylogenetic tree was generated using amino acid sequences identified by BlastP searches against the NCBI nr database, with the DepB sequence used as the query. Multiple sequence alignment was performed using Clustal Omega, and the tree was inferred using the Maximum Likelihood method; Figure S4: Phylogenetic analysis of the SPG protein from *Gossipyum hirsutum* and homologous sequences from diverse fungal and plant species. The phylogenetic tree was generated using amino acid sequences identified by BlastP searches against the NCBI nr database, with the DepA sequence used as the query. Multiple sequence alignment was performed using Clustal Omega, and the tree was inferred using the Maximum Likelihood method; Figure S5: Allosteric communication within the dimeric Fhb7 enzyme evaluated using the AlloSigMA algorithm to predict the propagation of energetic perturbations upon DON binding. (A) Per-residue free-energy profiles (Δh) for chains A and B, showing stabilizing (negative Δh) and destabilizing (positive Δh) responses along each polypeptide chain. Residues constituting the DON-binding pocket are indicated by gray circles. (B) Structural representation of the Fhb7 dimer colored according to the calculated energetic response, where blue indicates stabilization and red denotes destabilization. (C) Two-dimensional Δh matrix illustrating residue-to-residue energetic communication across the dimer. Figure S6: Comparative analysis of the surface electrostatics and active-site pocket architecture of SPG glyoxalase enzymes from *Gossypium hirsutum* and *Triticum aestivum*. Panels A and B display the electrostatic potential maps projected onto the molecular surfaces of SPG from *Gossypium* and the glyoxalase enzyme from *Triticum*, respectively. Positively charged regions are depicted in blue, negatively charged regions in red, and neutral surfaces in white. The *Gossypium* SPG glyoxalase enzyme exhibits a larger and more electropositive catalytic pocket compared to the *Triticum* homolog, suggesting potential differences in substrate accessibility and binding affinity; Figure S7: Coevolutionary analysis of the SPG glyoxalase enzyme from *Gossypium hirsutum* using the MISTIC2 server. The figure integrates sequence conservation and mutual information (MI)-based coevolutionary couplings derived from multiple sequence alignments of SPG glyoxalase homologs. (A) Sequence logo representation depicting the amino acid conservation across the SPG glyoxalase protein family. (B) Circos plot depicting the significant coevolutionary connections between residue pairs in SPG protein family, as measured by MI scores. Thicker and more intense arcs represent stronger coevolutionary couplings. (C) Connectivity network of residues with high conservation and/or strong coevolutionary connectivity, highlighting potential structurally or functionally important positions; Table S1: numerical data of the MM-PBSA analysis results during the analysis of glutathione and DON binding to Sph7 enzyme dimer subunits; Table S2: coevolution analysis results of SPG protein as computed by MISTIC2 algorithm.
