# Supplementary figures and images for: Cross-Kingdom Enzymatic Strategies for Deoxynivalenol Detoxification: Computational Analysis of Structural Mechanisms and Evolutionary Adaptations
Source: Microorganisms. 2025 Oct 16;13(10):2384. doi: 10.3390/microorganisms13102384 (PMC12566506; doi:10.3390/microorganisms13102384)

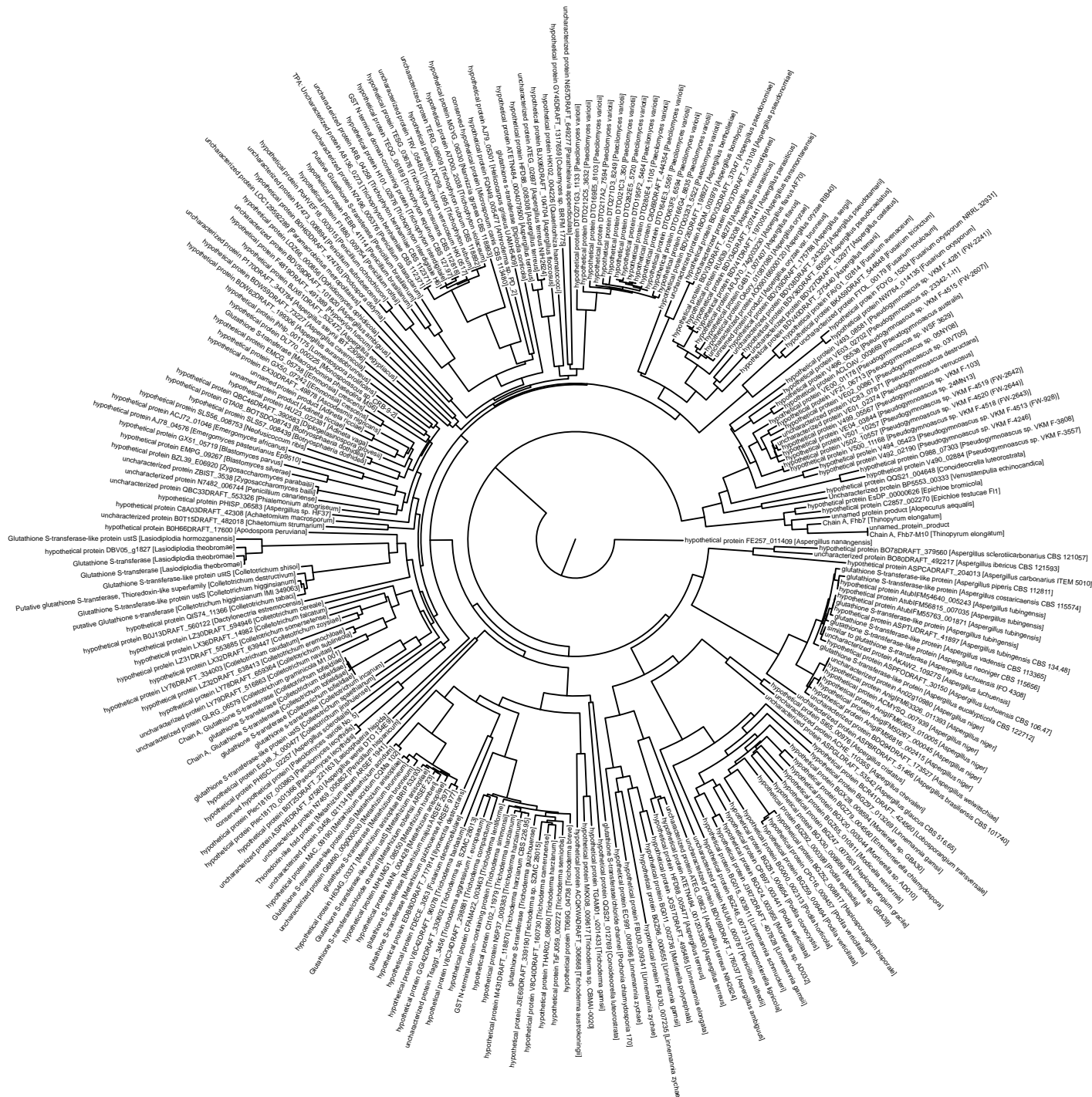

Supplement: Supplementary file 1 [file microorganisms-13-02384-s001.zip › Figure S1.pdf]

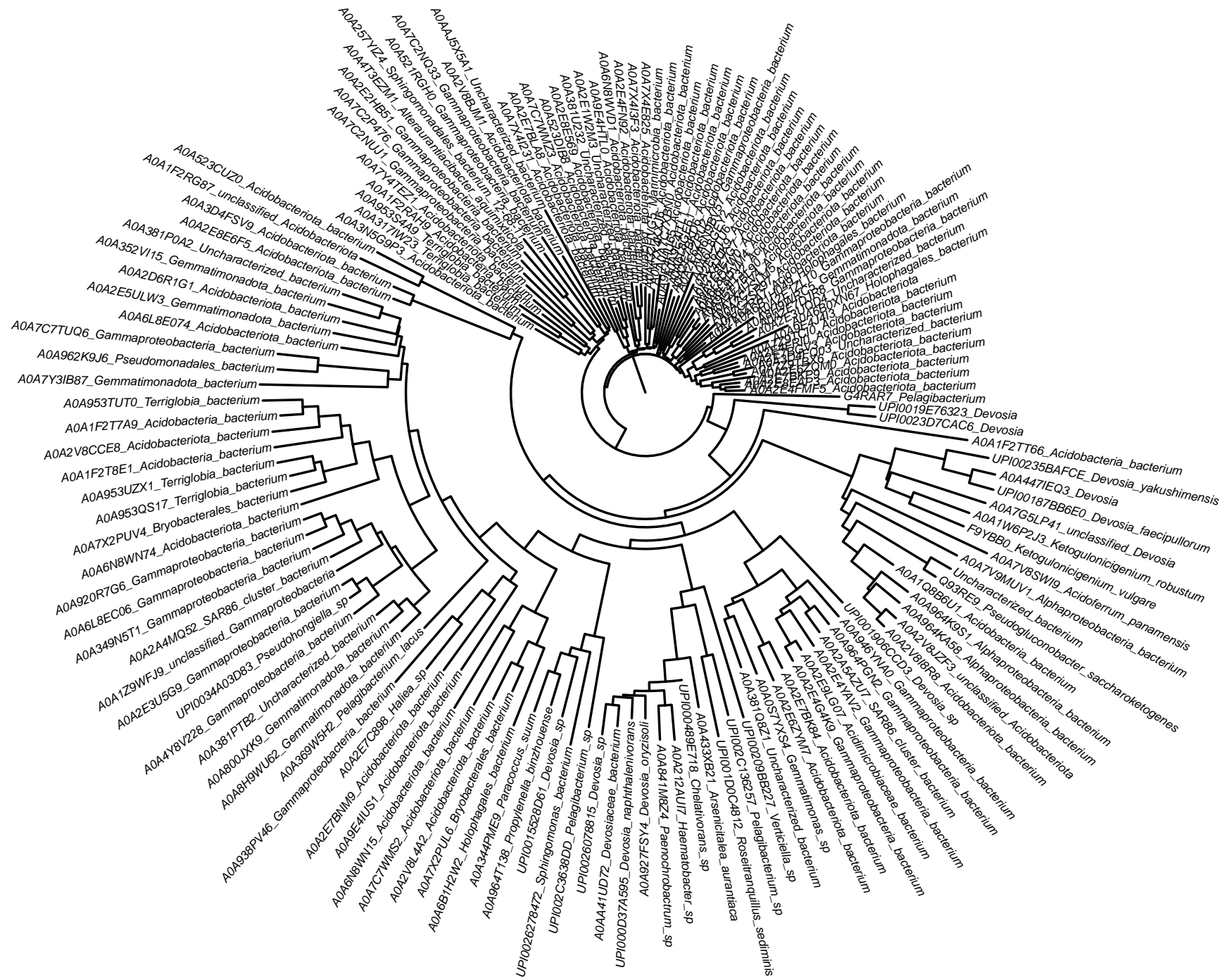

Supplement: Supplementary file 1 [file microorganisms-13-02384-s001.zip › Figure S2.pdf]

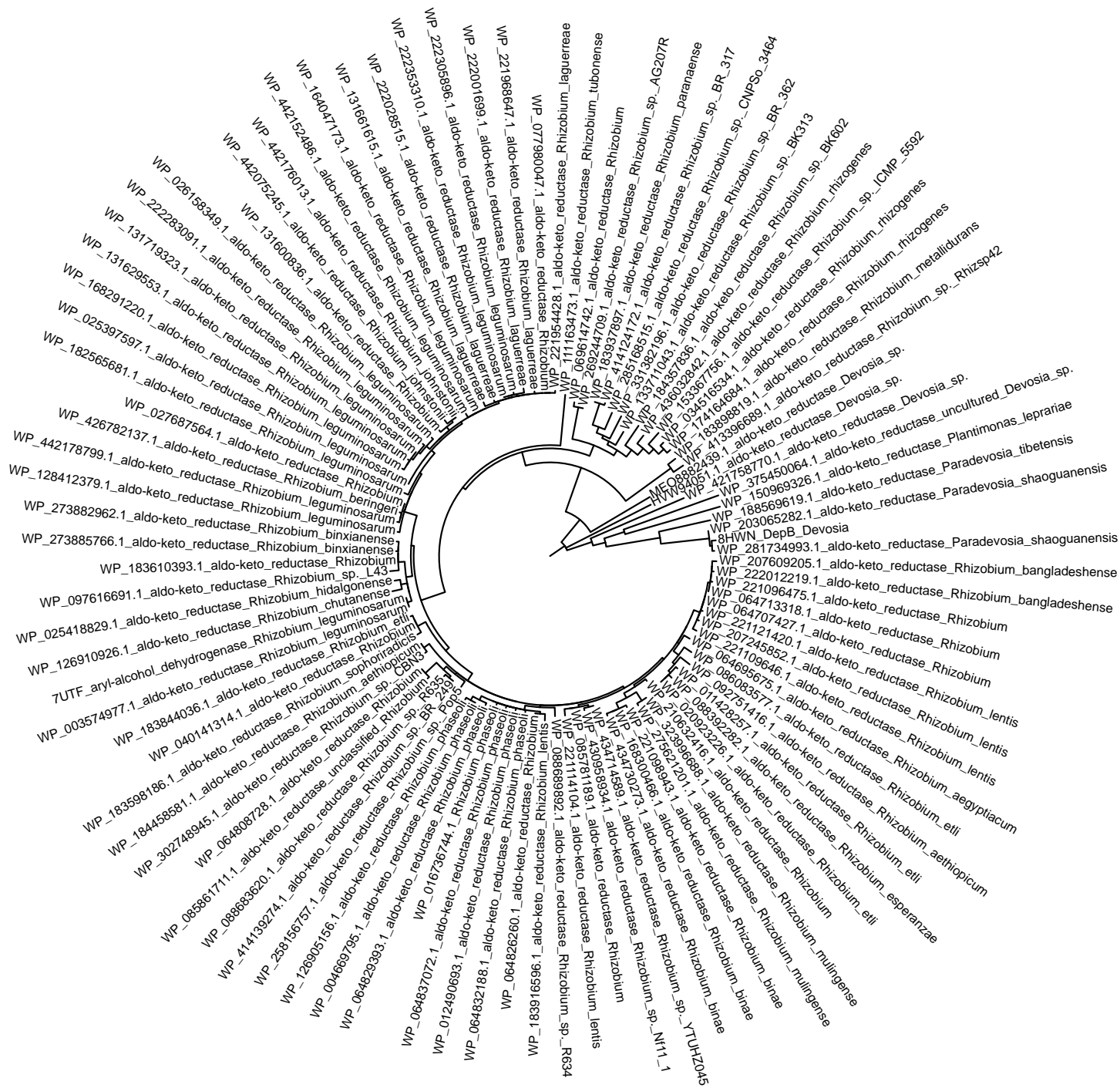

Supplement: Supplementary file 1 [file microorganisms-13-02384-s001.zip › Figure S3.pdf]

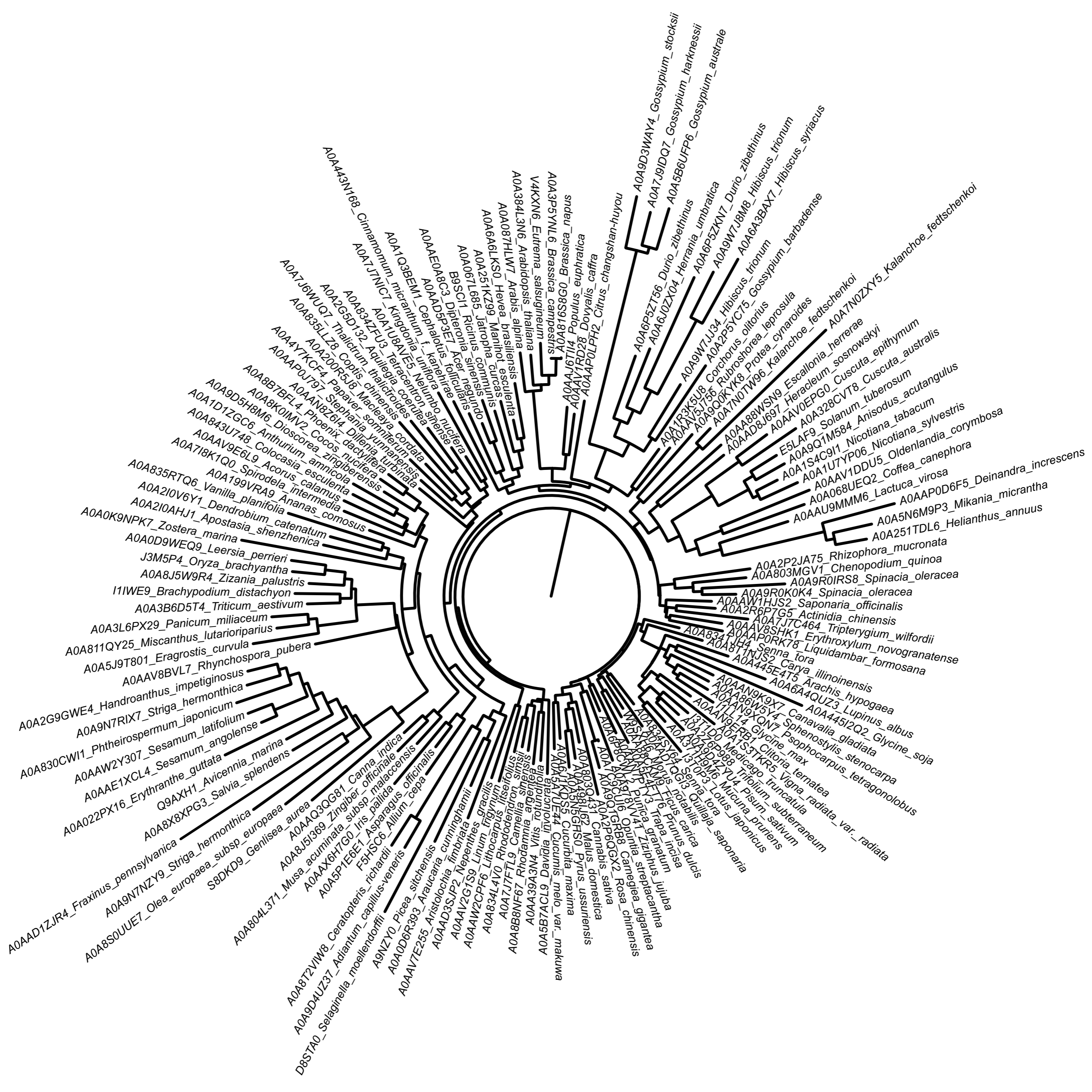

Supplement: Supplementary file 1 [file microorganisms-13-02384-s001.zip › Figure S4.pdf]

**A**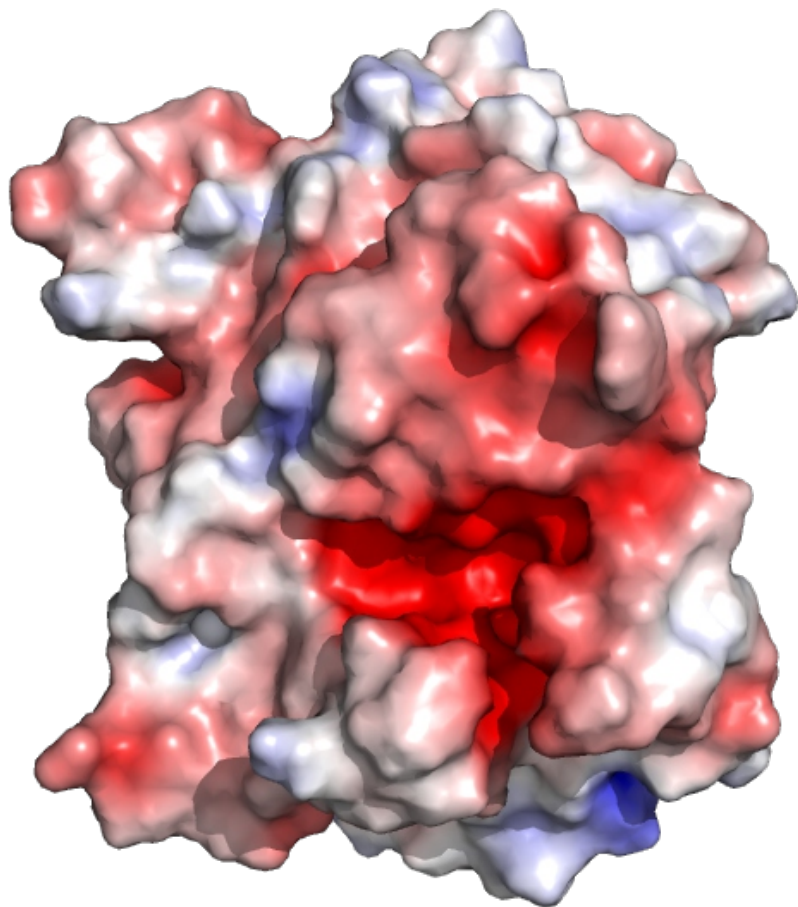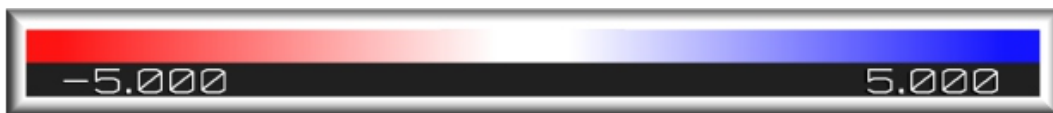

*Gossypium* SPG

Pocket volume : 2,281 Å<sup>3</sup>

**B**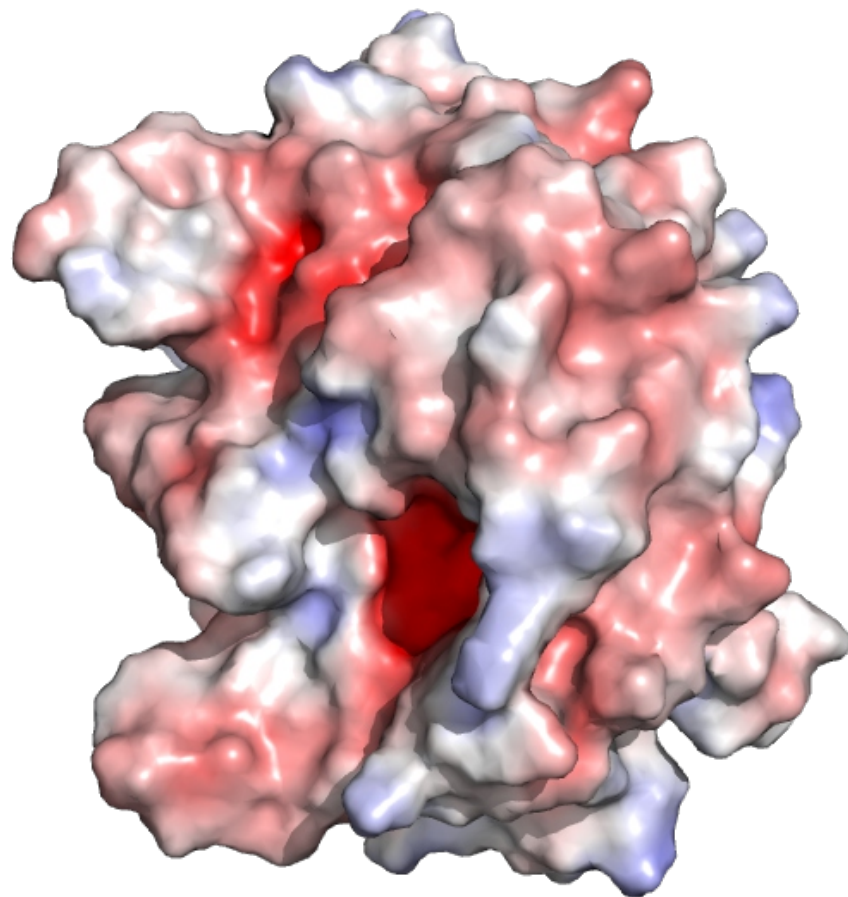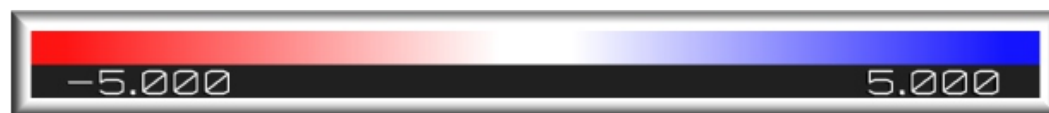

*Triticum* glyoxalase

Pocket area : 1,363 Å<sup>3</sup>

Supplement: Supplementary file 1 [file microorganisms-13-02384-s001.zip › Figure S6.pdf]

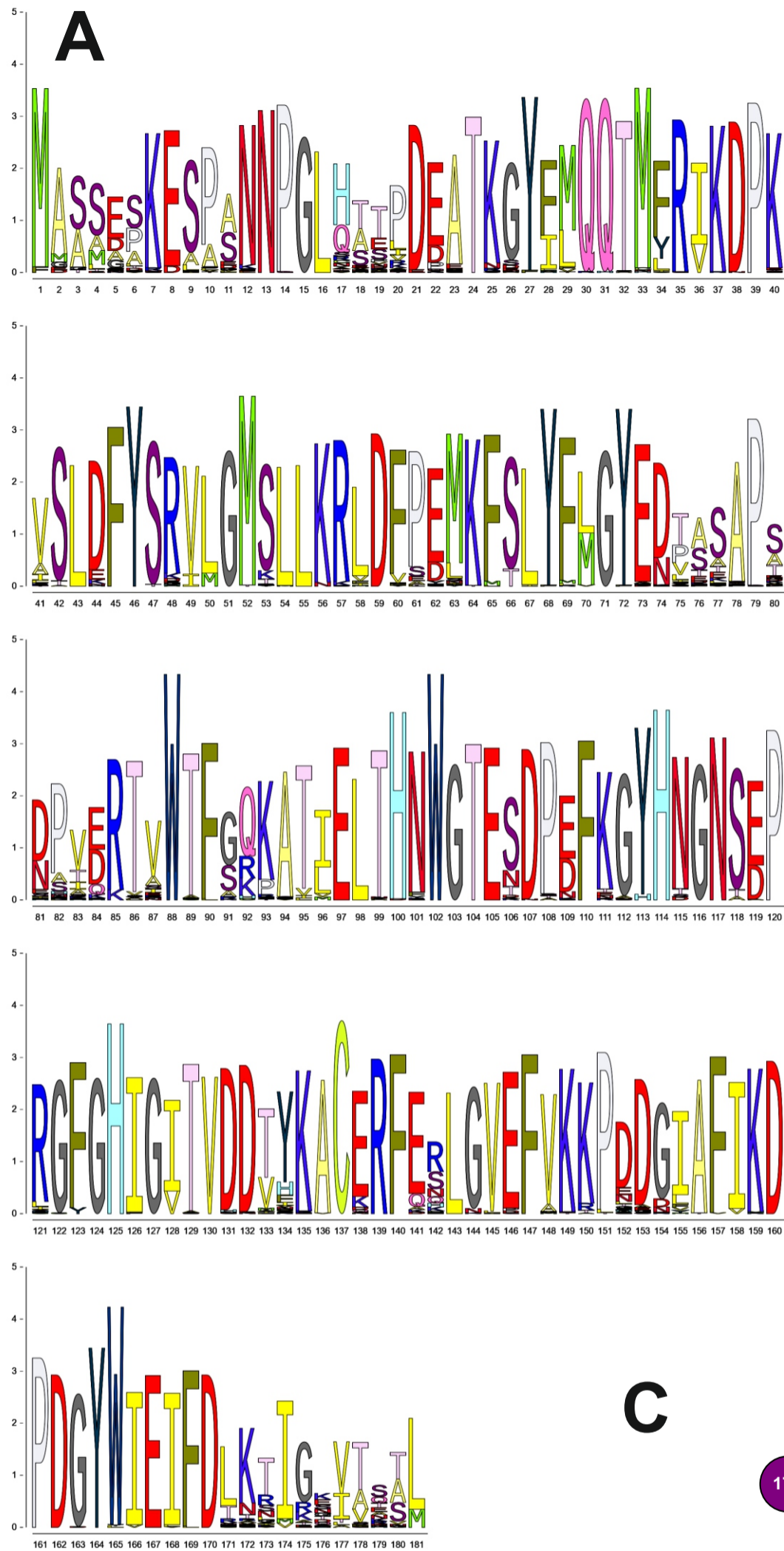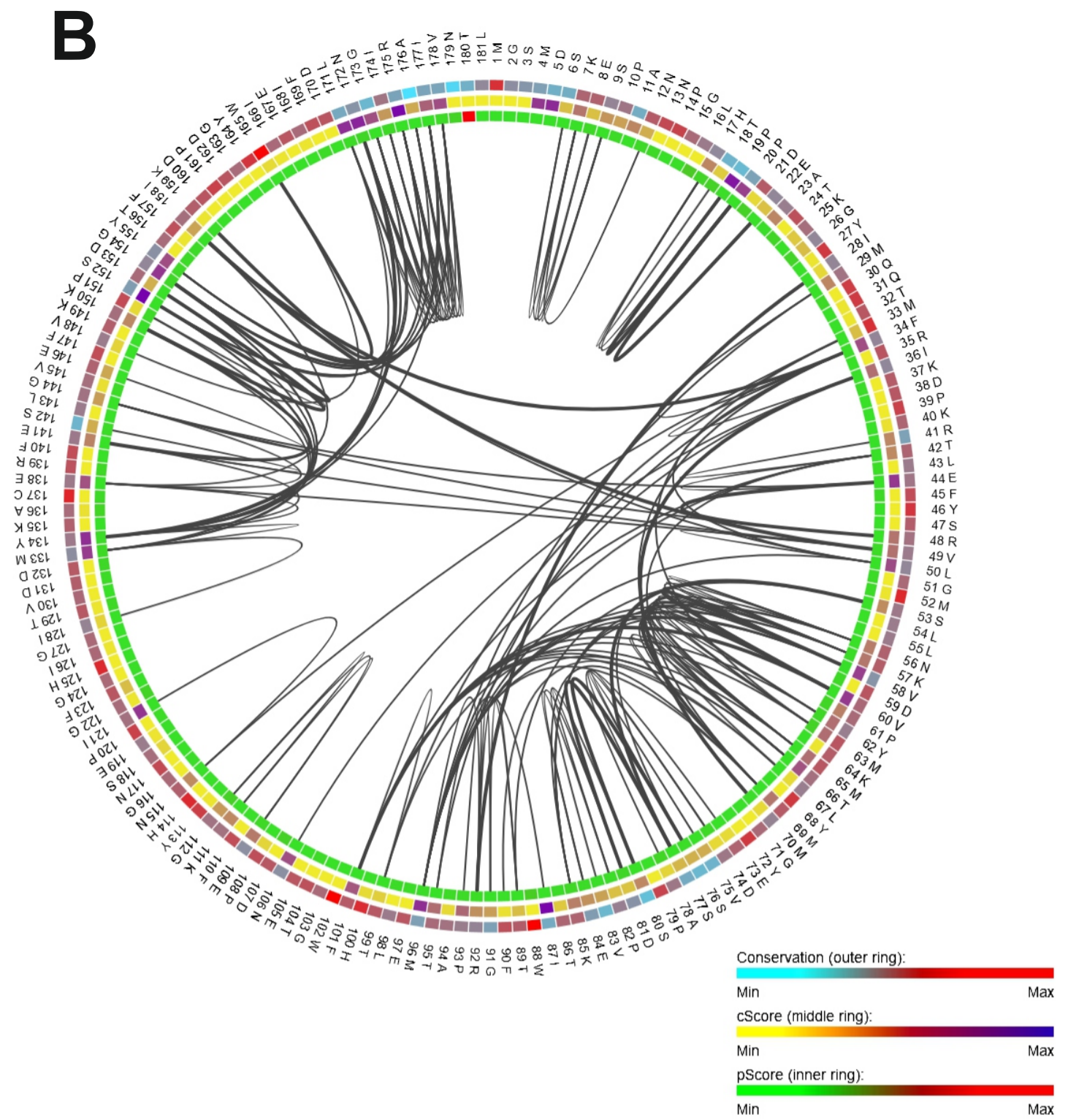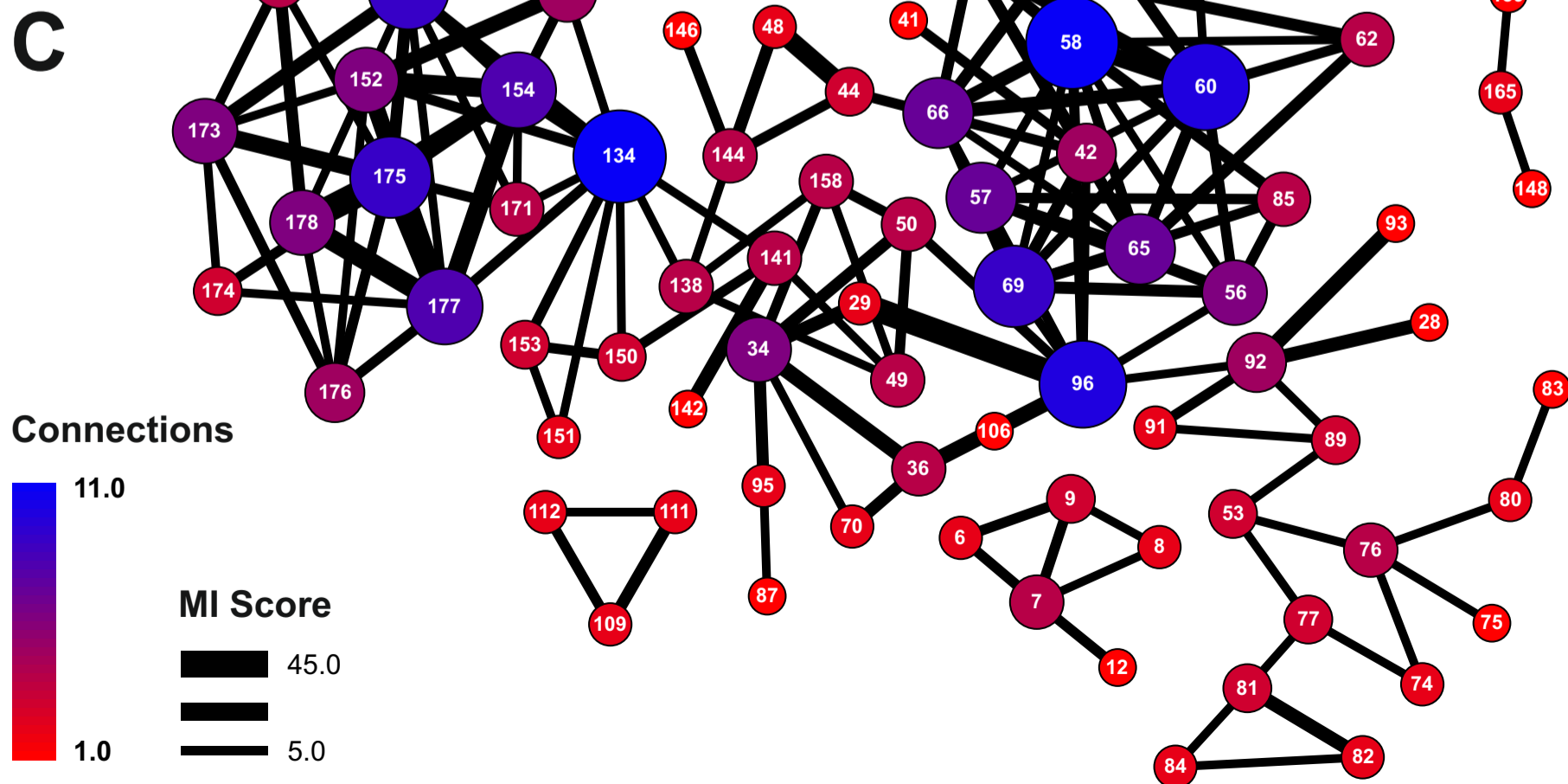

Supplement: Supplementary file 1 [file microorganisms-13-02384-s001.zip › Figure S7.pdf]

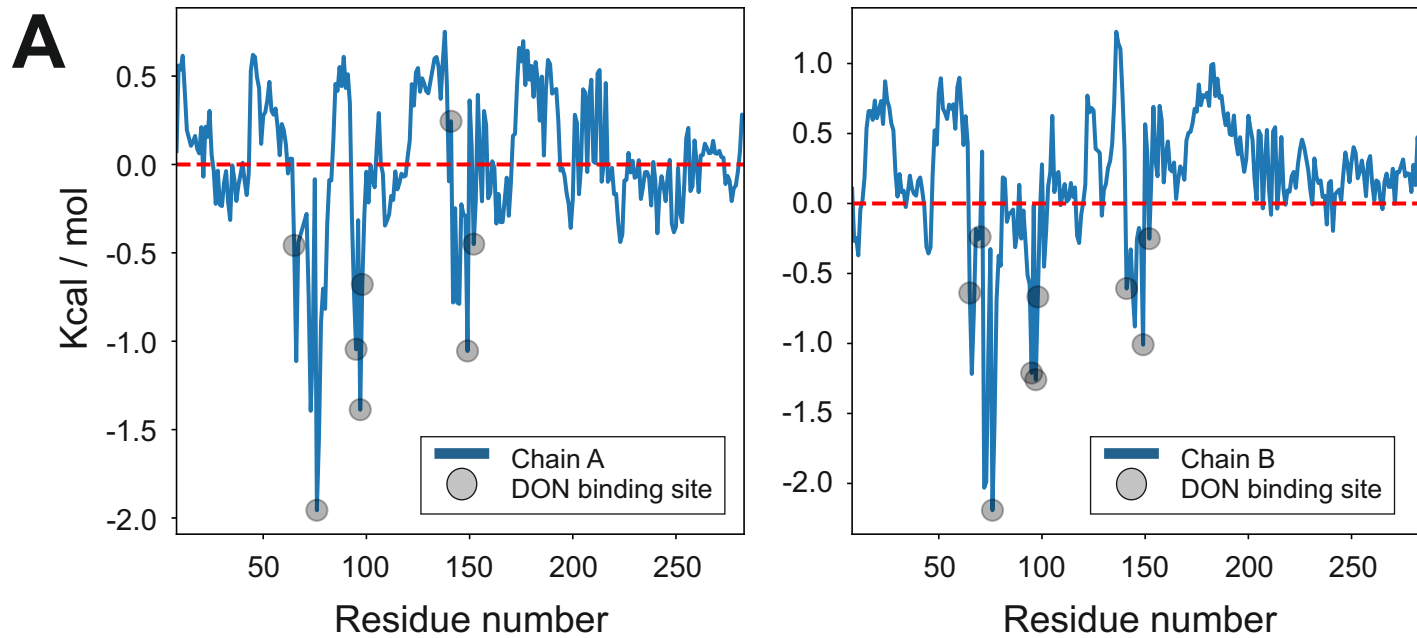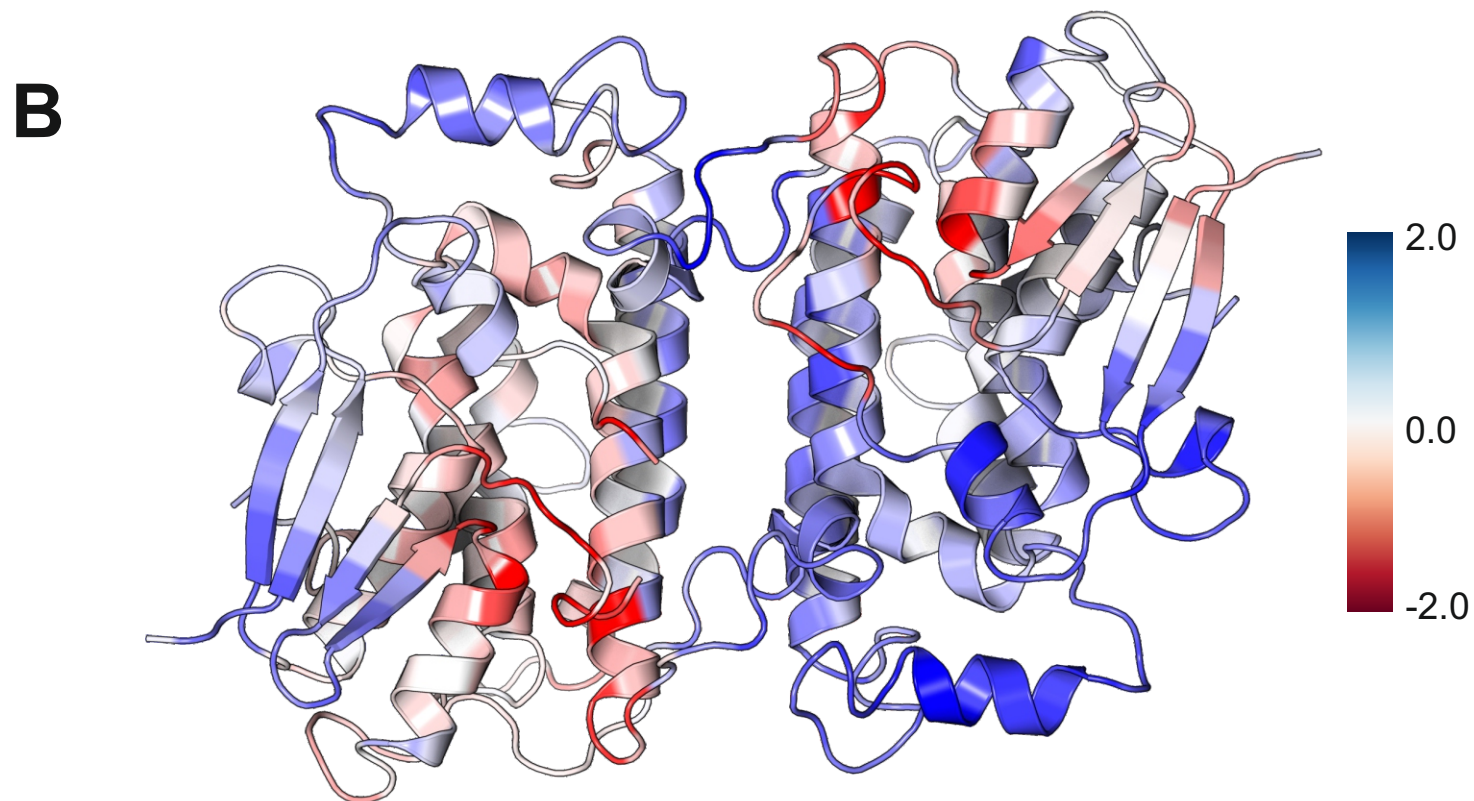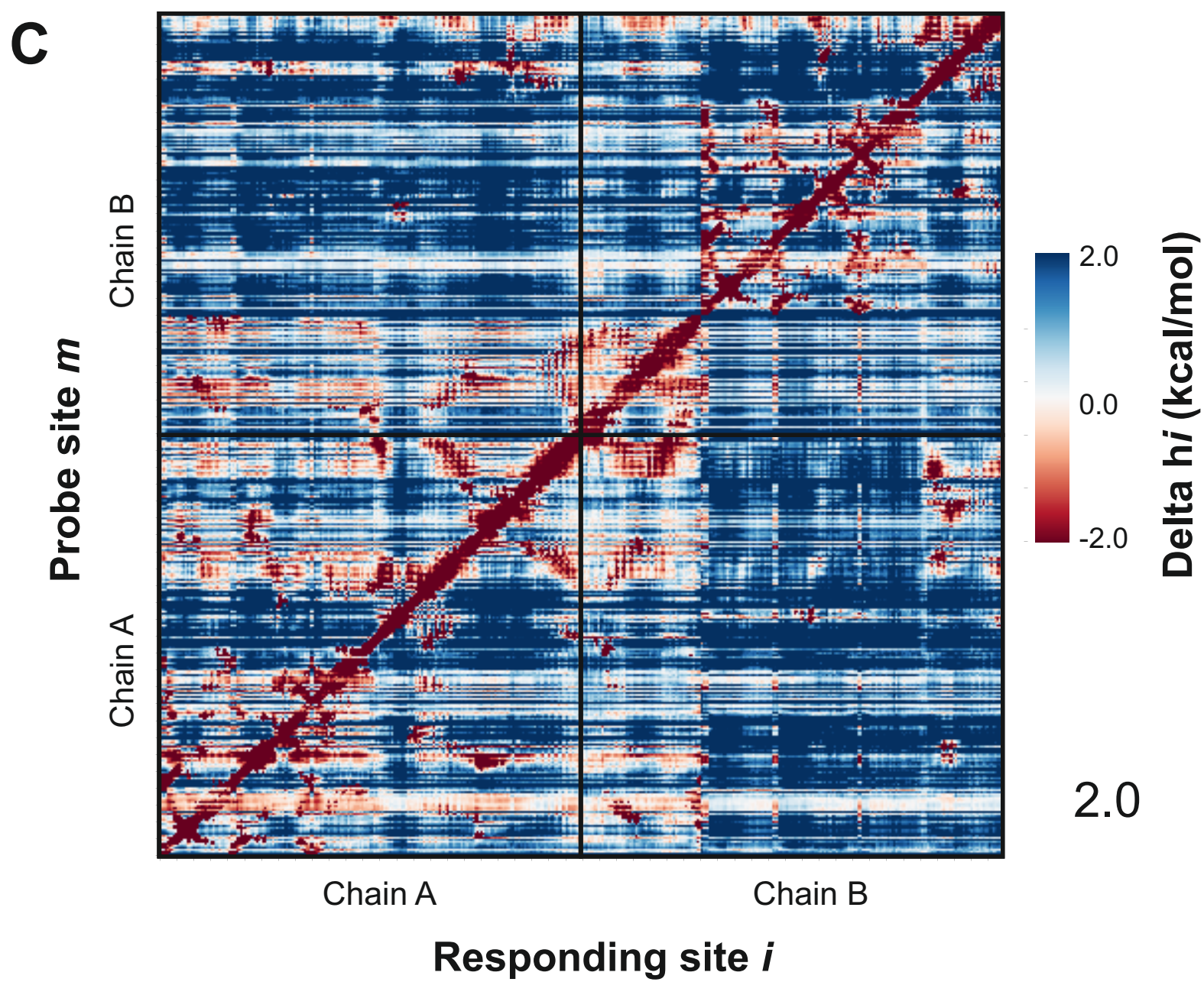

Supplement: Supplementary file 1 [file microorganisms-13-02384-s001.zip › Figure S5.pdf]
